# Associations of cigarette smoking with psychiatric disorders: evidence from a two-sample Mendelian randomization study

**DOI:** 10.1038/s41598-020-70458-4

**Published:** 2020-08-14

**Authors:** Shuai Yuan, Honghui Yao, Susanna C. Larsson

**Affiliations:** 1 Unit of Cardiovascular and Nutritional Epidemiology, Institute of Environmental Medicine, Karolinska Institutet, Nobelsväg 13, 17177 Stockholm, Sweden; 2 Department of Learning, Informatics, Management and Ethics, Karolinska Institutet, Stockholm, Sweden; 3 Department of Surgical Sciences, Uppsala University, Dag Hammarskjölds Väg 14B, 75185 Uppsala, Sweden

**Keywords:** Psychology, Human behaviour

## Abstract

We conducted a two-sample Mendelian randomization study to determine the association of smoking initiation with seven psychiatric disorders. We used 353 independent single-nucleotide polymorphisms associated with cigarette smoking initiation as instrumental variables at genome-wide significance threshold (*p* < 5 × 10^−8^) from a recent genome-wide association study in 1,232,091 European-origin participants. Summary-level data for seven psychiatric disorders, including anxiety, bipolar disorder, insomnia, major depressive disorder, posttraumatic stress disorder, suicide attempts, and schizophrenia, was obtained from large genetic consortia and genome-wide association studies. The odds ratios of genetically predicted smoking initiation were 1.96 for suicide attempts (95% CI 1.70, 2.27; *p* = 4.5 × 10^−20^), 1.69 for post-traumatic stress disorder (95% CI 1.32, 2.16; *p* = 2.5 × 10^−5^), 1.54 for schizophrenia (95% CI 1.35, 1.75; *p* = 1.6 × 10^−10^), 1.41 for bipolar disorder (95% CI 1.25, 1.59; *p* = 1.8 × 10^−8^), 1.38 for major depressive disorder (95% CI 1.31, 1.45; *p* = 2.3 × 10^−38^), 1.20 for insomnia (95% CI 1.14, 1.25; *p* = 6.0 × 10^−14^) and 1.17 for anxiety (95% CI 0.98, 1.40; *p* = 0.086). Results of sensitivity analyses were consistent and no horizontal pleiotropy was detected in MR-Egger analysis. However, the associations with suicide attempts, schizophrenia, bipolar disorder, and anxiety might be related to possible reverse causality or weak instrument bias. This study found that cigarette smoking was causally associated with increased risks of a number of psychiatric disorders. The causal effects of smoking on suicide attempts, schizophrenia, bipolar disorder and anxiety needs further research.

## Introduction

Psychiatric disorder, known as mental disorder or mental illness, has become an important health issue due to its high morbidity and mortality risk^1^. It was estimated that over 8 percentage of the global population had at least one major mental disorder in 2015^2^ and global disease burden of mental illness ranked the second, accounting for around 11.2% of disability-adjusted life years in 2013^3^.

Cigarette smoking has been proposed as a risk factor for the majority of psychiatric disorder^4–6^. Observational studies have found that cigarette smoking is associated with an increased risk of a number of psychiatric disorders, including suicide, major depressive disorder, and bipolar disorder, etc^7,8^. In addition, several meta-analysis of prospective studies revealed a dose–response relationship between smoking and the risk of suicide and schizophrenia^9–12^. However, whether these observed associations are causal remains unclear since the findings in observational studies are prone to be biased by reverse causality (prevalent nicotine dependence among psychiatric patients, such as schizophrenic patients^13^), residual confounding and misclassification.

Genetic variants can be used as instrumental variables to assess the causal exposure-outcome association, which is known as Mendelian randomization (MR) analysis. This approach can reduce confounding and overcome reverse causality because genetic variants are randomly distributed at conception and cannot be affected by disease status. We conducted a two-sample MR study to investigate whether smoking initiation is causally associated with seven major psychiatric disorders. Given that previous studies have suggested a protective effect of smoking against depression^14^, we additionally assessed the reverse influence of depression on smoking initiation.

## Methods

### Study design

**Figure 1:**
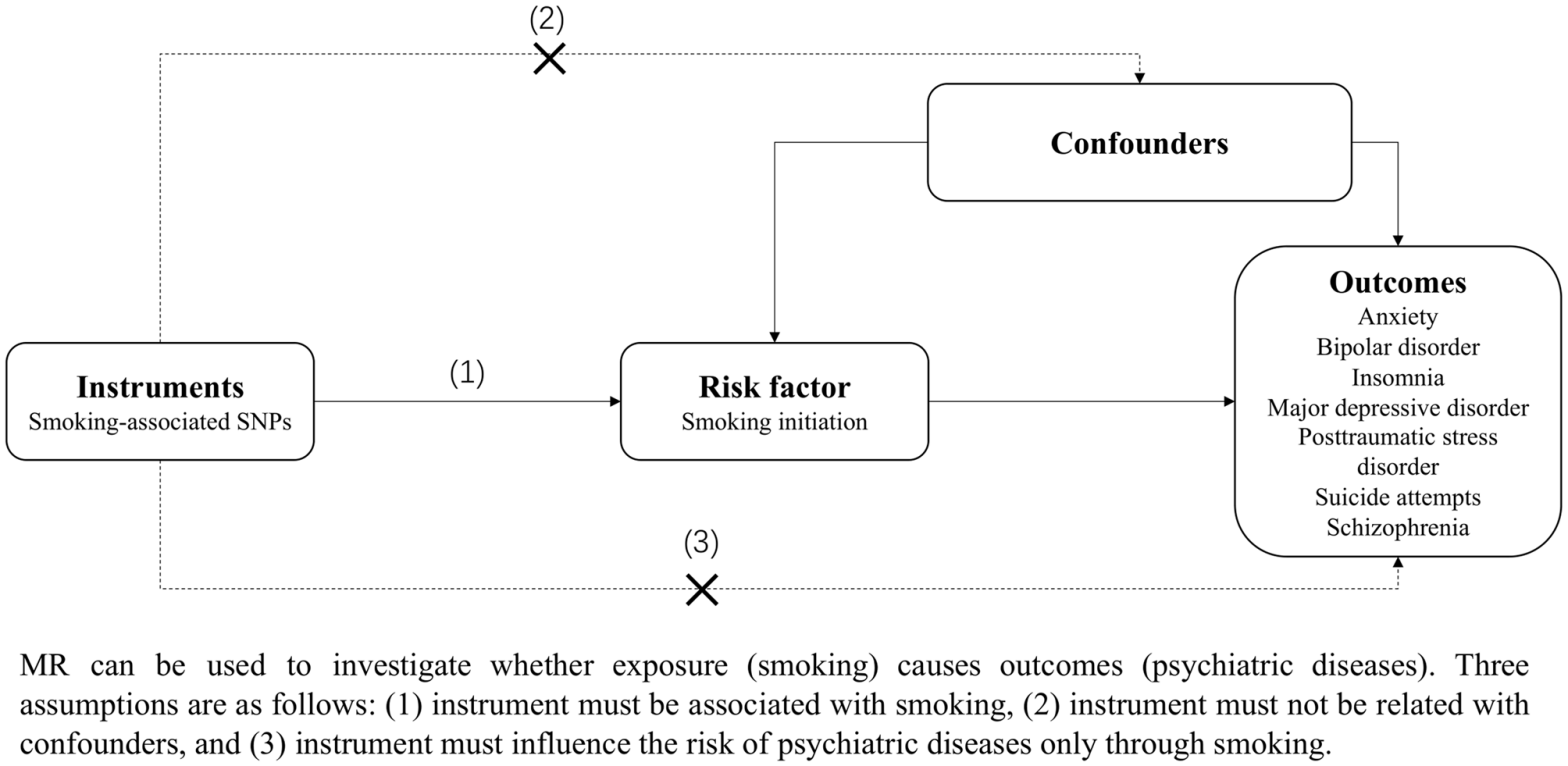
Schematic diagram of the Mendelian randomization assumptions. The MR design can be used to investigate whether a modifiable risk factor (e.g., smoking) is causally related to the outcome (e.g., psychiatric disease). Three assumptions of the present MR study are: (1) the genetic instrument must be associated with smoking, (2) the instrumental variables must not be related to any confounders, and (3) the instrument must influence the risk of psychiatric diseases only through smoking and not through any direct causal pathway.

The present MR analysis sets basis at three key assumptions: (1) the genetic variants used as instrumental variables should be tightly associated with smoking; (2) the genetic variants used instrumental variables should not associated with any confounders of the association between smoking and psychiatric disorders, and (3) the genetic variants should only affect the risk of the psychiatric disorders via smoking (Fig. 1)^15^. In this study, we included seven major psychiatric disorders, including anxiety, bipolar disorder, insomnia, major depressive disorder, posttraumatic stress disorder, suicide attempts, and schizophrenia^16–22^, using the summary-level data from publicly available genome-wide association studies (GWAS). Individual studies included in the GWAS datasets had been approved by an ethical review board. No individual-level data were used in the present MR study.

### Instrumental variable selection

For smoking initiation, we selected 378 single-nucleotide polymorphisms (SNPs) as instrumental variables at the genome-wide significance threshold (*p* < 5 × 10^−8^) from a GWAS in 1,232,091 European-origin participants^23^. We excluded SNPs with linkage disequilibrium (R^2^ > 0.1), leaving 353 independent instrumental variables for smoking initiation. Genetic principle components, population stratification and relatedness of participants were adjusted for in the GWAS. Cigarette initiation was defined as smoking every day for at least a month, smoking more than 100 cigarettes over total life or smoking regularly. Detailed information about SNPs is shown in Supplementary Table 1.

**Table 1:** Characteristics of included genome-wide association studies for smoking initiation and psychiatric disorders.

Exposure/outcome	Cases, No	Controls, No	Population	Used SNPs, No.^b^	Power^c^	Source	Consortium
**Exposure**							
Smoking initiation	NA	1,232,091^a^	European	NA	NA	Liu et al.^14^	GSCAN
**Outcome**							
Anxiety	7,016	14,745	European	348	0.45	Otowa et al.^15^	Psychiatric Genomics Consortium
Bipolar disorder	20,352	31,358	European	352	1.00	Stahl et al.^16^	Psychiatric Genomics Consortium
Insomnia	397,959	933,051	European	344	1.00	Jansen et al.^17^	CNCR
Major depressive disorder	170,756	329,443	European	347	1.00	Stahl et al.^18^	UK Biobank
Posttraumatic stress disorder	30,000	170,000	Mix	351	1.00	Nievergelt et al.^19^	Psychiatric Genomics Consortium
Suicide attempts	6,024	44,240	European	304	1.00	Erlangsen et al.^20^	iPSYCH
Schizophrenia	33,426	54,065	European	350	1.00	Psychiatric Genomics Consortium^21^	Psychiatric Genomics Consortium

CNCR, Center for Neurogenomics and Cognitive Research; GSCAN, GWAS and Sequencing Consortium of Alcohol and Nicotine use; SNP, single-nucleotide polymorphism.

^a^The number represents the number of total population in the GWAS analysis for smoking initiation.

^b^The number of all SNPs reaching the genome-wide significance level for smoking initiation is 378, leaving 353 SNPs proposed as instrumental variables after LD exclusion (R^2^ > 0.1).

^c^We assumed that the used SNPs for smoking initiation in the analysis of each outcome explained around 2% phenotypic variance. Power calculation was based on a web-tool: https://cnsgenomics.com/shiny/mRnd/.

### Source of outcomes

Summary-level genetic data for seven psychiatric disorders were obtained from large-scale GWASs or genetic consortia^16–22^. Descriptions of outcome sources, such as the number of controls and cases, population structure and dataset source, are presented in Table 1. The definitions of included disorders are listed in Supplementary Table 2.

**Table 2:** Association of genetically predicted smoking initiation with psychiatric disorder in sensitivity analysis.

Psychiatric disorder	Weighted median			MR-PRESSO^a^			MR-Egger			MR-Egger (SIMEX)			I^2^_GX_	*P* for pleiotropy^b^
	OR	95% CI	*p*	OR	95% CI	*p*	OR	95% CI	*p*	OR	95% CI	*p*		
Suicide attempts	1.99	1.63, 2.46	2.1 × 10^−12^	1.97	1.70, 2.27	7.1 × 10^−18^	1.42	0.78, 2.61	0.310	1.63	0.79, 3.40	0.188	61%	0.29
Post-traumatic stress disorder	1.79	1.24, 2.57	0.002	1.70	1.32, 2.16	3.1 × 10^−5^	1.55	0.54, 4.45	0.419	1.90	0.33, 10.8	0.468	59%	0.86
Schizophrenia	1.42	1.24, 1.61	2.3 × 10^−7^	1.55	1.38, 1.73	5.9 × 10^−13^	2.20	1.25, 3.92	0.007	3.32	1.20, 9.14	0.021	58%	0.20
Bipolar disorder	1.54	1.34, 1.76	1.3 × 10^−10^	1.49	1.34, 1.67	4.1 × 10^−12^	1.79	1.07, 3.02	0.027	2.23	0.96, 5.13	0.062	59%	0.35
Major depressive disorder	1.38	1.31, 1.44	6.4 × 10^−38^	1.40	1.34, 1.46	3.4 × 10^−42^	1.19	0.97, 1.47	0.101	1.28	0.93, 1.75	0.125	59%	0.15
Insomnia	1.22	1.16, 1.29	9.1 × 10^−14^	1.20	1.14, 1.25	1.6 × 10^−13^	1.08	0.89, 1.32	0.420	1.13	0.76, 1.67	0.557	60%	0.32
Anxiety	1.17	0.90, 1.51	0.234	1.17	0.98, 1.40	0.086	1.25	0.55, 2.80	0.597	1.32	0.42, 4.15	0.627	55%	0.88

CI, confidence interval; I^2^GX, I^2^ statistic for the SNP-exposure (GX) effect; MR-Egger, Mendelian randomization-Egger regression; MR-Egger (SIMEX), Mendelian randomization-Egger with simulation extrapolation; MR-PRESSO, Mendelian randomization pleiotropy residual sum and outlier; OR, odds ratio.

^a^We detected 16, 8, 18 and 4 outliers in the MR-PRESSO analysis of schizophrenia, bipolar disorder, major depressive disorder and insomnia.

^b^*P* value for intercept in the MR-Egger analysis.

### Statistical analyses

The random-effects inverse-variance weighted method was used as the main analysis due to the most precise estimation it can provide^24^. However, because the inverse-variance weighted method is sensitive to invalid instrumental variables and pleiotropy^25^, several sensitivity analyses were additionally performed, including weighted median, MR Egger, MR-Egger-SIMEX and MR-PRESSO. The weighted median approach can provide a consistent estimate if more than 50% of weight comes from valid instrumental variables^25^. The MR-Egger regression is a technique to detect and correct for horizontal pleiotropy albeit with low power^26^. MR-Egger-SIMEX can provide estimation with adjustment for dilution of the MR-Egger estimate using the simulation extrapolation (SIMEX) method, as a supplementary tool for MR-Egger when I^2^_GX_ < 90%^26^. The MR pleiotropy residual sum and outlier (MR-PRESSO) method can generate empirical distribution of causal estimates by bootstrap and correct for horizontal pleiotropy via outlier removal^27^. Heterogeneity was assessed by I^2^ and Cochran’s Q value in the inverse-variance weighted model and Rucker’s Q’ value in MR-Egger regression. Compared with Cochran’s Q value, a lower Rucker’s Q’ value indicates that the MR-Egger method provides a model with a better fit for examining the particular association. Given that previous studies suggested a protective effect of smoking against depression^14^, we conducted a reverse MR analysis to assess the influence of having depression on smoking initiation.

To test the direction of causations, we used the MR Steiger directionality^28^ test to determine whether the observed associations were directionally causal. The rationale of this approach is to compare the variances explained by used SNPs in the exposure and outcomes. If the used instrumental variables explained more variance in smoking initiation than included psychiatric outcomes, the established associations could be directionally reliable. We calculated F statistics^29^ to examine the weak instrument bias using following formula: F = ((n − k − 1)/k) * (R^2^/(1 − R^2^)) where n, k and R^2^ indicates sample size, number of instrumental variables and variance explained by used SNPs, respectively. A generally quoted criterion is that an instrument is weak if the F statistic is less than 10. To visualize the associaitons and check the assumptions of MR, MR scator plots using three analyses were drawn. The summary statistics data across datasets were harmonized so that the effect allele reflected the allele associated with an increased probability of lifetime smoking initiation. The odds ratios (ORs) of psychiatric disorders with 95% confidence intervals (CIs) represent the increase of one standard deviation in the prevalence of smoking initiation. The power was calculated based on a web-tool^30^. All *p* values were two-sided. These analyses in the present study were performed using the mrrobust package in Stata/SE 15.0 (StataCorp. 2017. Stata Statistical Software: Release 15. College Station, TX: StataCorp LLC.) and TwoSampleMR package^31^ in R Software 3.6.0 (R Core Team. R Foundation for Statistical Computing. Vienna, Austria. 2019. https://www.R-project.org). We interpreted the findings based on the strength and consistency of the associations in the main and sensitivity analyses rather than defining the statistical significance threshold based on *p* values^32^.

## Results

**Figure 2:**
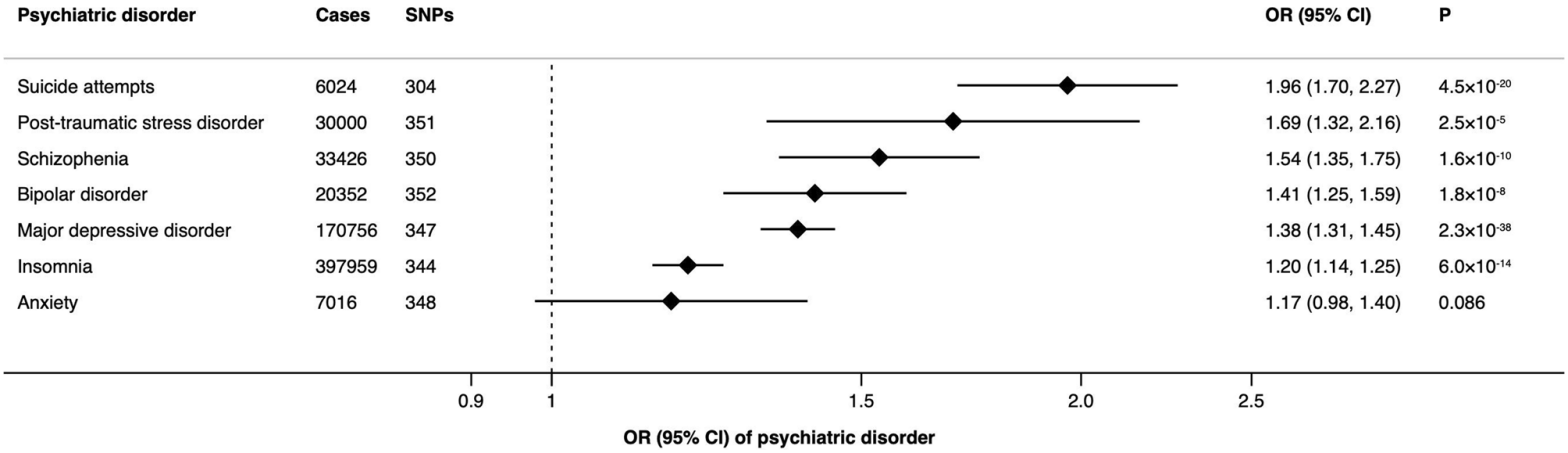
Associations of genetically predicted smoking initiation with psychiatric disorders using inverse-variance weighted model. CI indicates confidence interval; OR, odds ratio; SNPs, single-nucleotide polymorphisms. Estimates were estimated using the mrrobust package in Stata/SE 15.0 (StataCorp. 2017. Stata Statistical Software: Release 15. College Station, TX: StataCorp LLC.).

The associations between cigarette smoking initiation and psychiatric disorders are shown in Fig. 2. Genetically predicted smoking initiation was associated with higher odds of all seven included psychiatric disorders. The odds ratios were 1.96 (95% CI 1.70, 2.27; *p* = 4.5 × 10^−20^) for suicide attempts, 1.69 (95% CI 1.32, 2.16; *p* = 2.5 × 10^−5^) for post-traumatic stress disorder, 1.54 (95% CI 1.35, 1.75; *p* = 1.6 × 10^−10^) for schizophrenia, 1.41 (95% CI 1.25, 1.59; *p* = 1.8 × 10^−8^) for bipolar disorder, 1.38 (95% CI 1.31, 1.45; *p* = 2.3 × 10^−38^) for major depressive disorder, 1.20 (95% CI 1.14, 1.25; *p* = 6.0 × 10^−14^) for insomnia and 1.17 (95% CI 0.98, 1.40; *p* = 0.086) for anxiety in the main analysis. Results remained consistent across sensitivity analyses albeit with larger CIs in weighted median and MR-Egger analyses (Table 2). We detected moderate to high heterogeneity in the analysis of bipolar disorder, schizophrenia, major depressive disorder and insomnia (Supplementary Table 3). However, there was no horizontal pleiotropy in any analysis (*p* for the MR-Egger intercept > 0.05). We observed I^2^_GX_ of around 60% in all analyses, indicating possible dilution in the MR-Egger estimation due to violation of the No Measurement Error assumption. After correcting for dilution, the magnitude of all associations in the MR-Egger-SIMEX analysis increased slightly (Table 2). We observed several Rucker’s Q’ values > 700 (*p* < 0.001), indicating that the MR-Egger approach did not provide a model with a better fit compared to the inverse-variance weighted method. Four to eighteen outliers were detected in the analysis of bipolar disorder, schizophrenia, major depressive disorder, and insomnia. After outlier removal, the significance and magnitude of all associations persisted in MR-PRESSO (Table 2).

**Figure 3:**
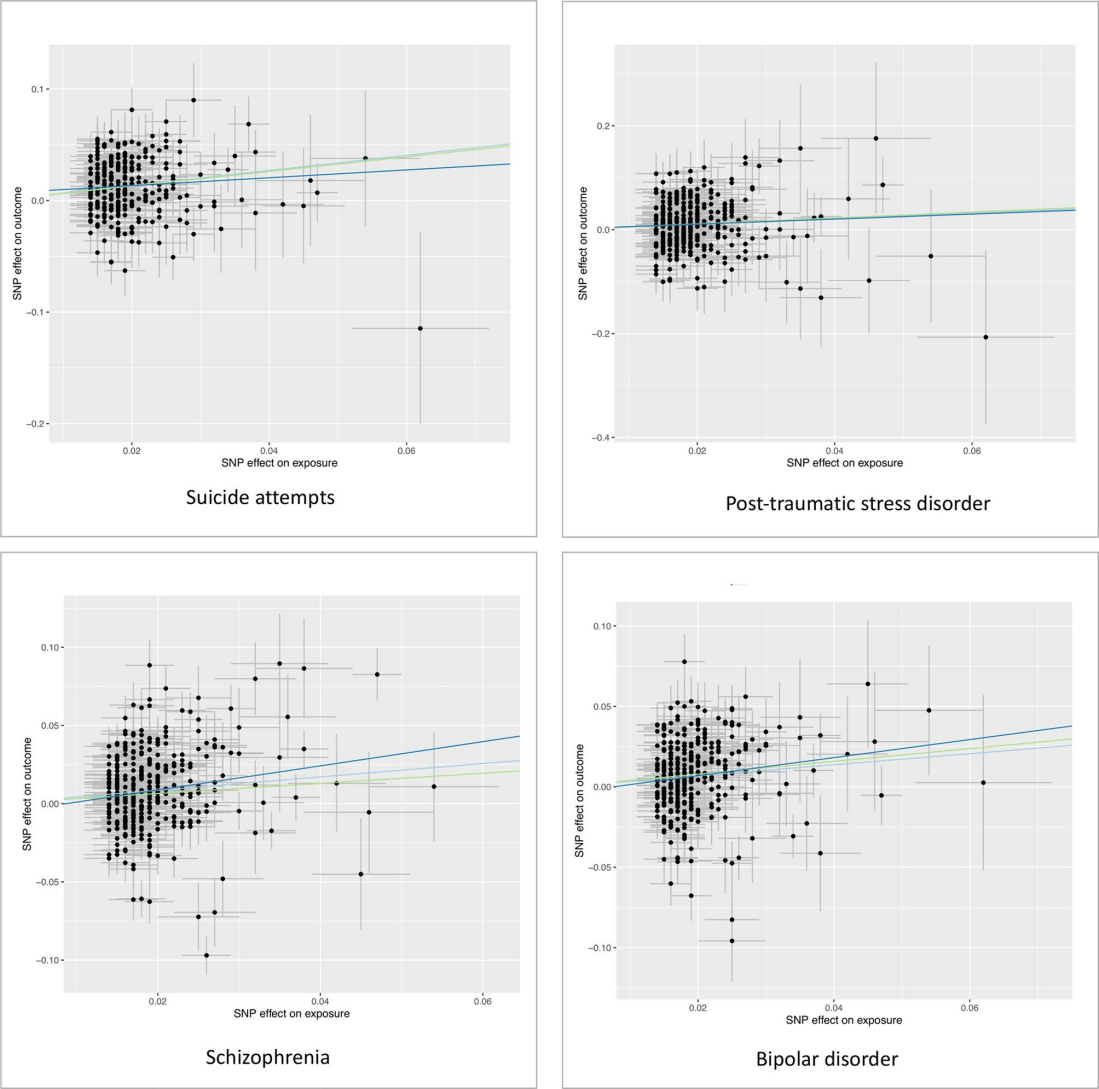

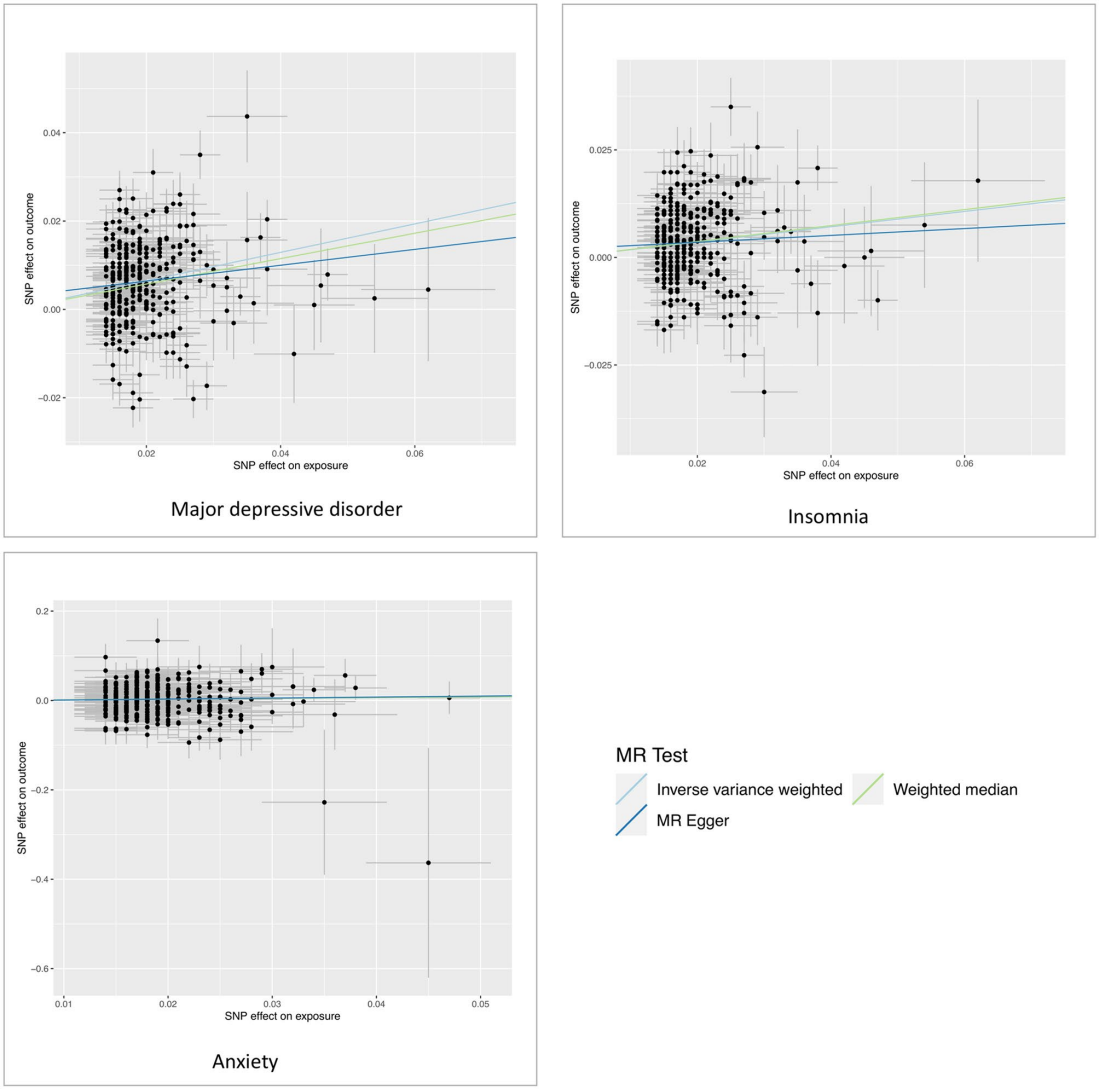
Scatter plots of the associations of smoking initiation with seven psychiatric diseases. The Scatter plots were constructed using TwoSampleMR package^31^ in R Software 3.6.0 (R Core Team. R Foundation for Statistical Computing. Vienna, Austria. 2019. https://www.R-project.org). Betas (SEs) and p values were 0.68 (0.07) and 4.6 × 10^−20^ in inverse-variance weigthed (IVW), 0.65 (0.10) and 1.8 × 10^−10^ in weighted median (WM), 0.35 (0.31) and 0.256 in MR-Egger for suicide attempts; 0.53 (0.12) and 2.2 × 10^−5^ in IVW, 0.56 (0.18) and 2.2 × 10^−3^ in WM, 0.49 (0.54) and 0.366 in MR-Egger for post-traumatic stress disorder; 0.43 (0.07) and 1.7 × 10^−10^ in IVW, 0.32 (0.07) and 9.5 × 10^−7^ in WM, 0.77 (0.29) and 0.008 in MR-Egger for schizophrenia; 0.34 (0.06) and 2.0 × 10^−8^ in IVW, 0.40 (0.07) and 2.0 × 10^−7^ in WM, 0.56 (0.26) and 0.035 in MR-Egger for bipolar disorder; 0.32 (0.02) and 1.8 × 10^−38^ in IVW, 0.29 (0.02) and 1.5 × 10^−34^ in WM, 0.18 (0.11) and 0.091 in MR-Egger for major depressive disorder; 0.18 (0.02) and 6.2 × 10^−14^ in IVW, 0.19 (0.03) and 4.8 × 10^−12^ in WM, 0.08 (0.10) and 0.430 in MR-Egger for insomnia; and 0.16 (0.09) and 0.085 in IVW, 0.16 (0.13) and 0.229 in WM, 0.22 (0.41) and 0.598 in MR-Egger for anxiety.

We had 100% power in all analyses, except for analysis of anxiety with a power of 45%. In the MR Steiger directionality test, the variance explained by included SNPs is larger in schizophrenia, bipolar disorder and anxiety compared with smoking initiation, suggesting that these observed associations might not be directionally causal. In addition, we found the weak instrument bias might exist in the associations for anxiety, bipolar disorder, suicide attempts and schizophrenia (F statistic < 10). Figure 3 shows the scatter plots for all analyzed associations. The causal associations of smoking with depression and insomnia appeared to be stable given a stronger effect on outcome comparted with exposure. However, other associations might be risked by reverse causation or weak instrument bias, which is also partly revealed in MR Steiger directionality tes. In the reserve MR analysis for depression, the liability to depression was associated with an increased risk of smoking initiation (OR = 1.11; 95% CI 1.02–1.22; *p* = 0.016). The result remained consistent in the weighted median method, but not in MR-Egger regression (Supplementary Table 4).

## Discussion

The findings of the present two-sample MR study demonstrated positive associations of smoking initiation with six psychiatric disorders, including suicide attempts, post-traumatic stress disorder, schizophrenia, bipolar disorder, major depressive disorder, and insomnia. There was a suggestive positive association between smoking initiation and anxiety. However, the associations for suicide attempts, schizophrenia, bipolar disorder and anxiety could not be determined due to possible reverse causality or weak instrument bias. There was a reverse association between the liability to depression and increased risk of smoking initiation.

Observational studies have revealed that smoking initiation and other smoking-related traits were associated with overall and specific psychiatric disorders^5–8,12^, which is supported by the results of the present MR study. Findings of meta-analyses of cohort, case–control and/or cross-sectional studies have shown that smoking is associated with an increased risk of suicide attempts^12,33^, post-traumatic stress disorder^33^, schizophrenia^34^ and major depressive disorder^35^ as well as bipolar disorder^36^. Several prospective studies also found an elevated risk of insomnia among smokers^37^, especially among heavy smokers^38^. Observational findings of the association between smoking and anxiety are inconsistent in terms of the directions of the associations^39^. A previous MR study reported no association between smoking heaviness and anxiety, but that study relied on a single instrumental variable for smoking^40^. The present MR study, which exploited 348 SNPs as instrumental variables, detected a possible modest positive association between smoking initiation and anxiety but had inadequate power.

The associations of smoking with depression, schizophrenia^41^, suicide ideation^42^ and bipolar disorder^36^ have been revealed in previous MR studies. The present study was based on more recent GWASs and comprehensively investigated the causal associations between smoking initiation and psychiatric traits. We confirmed established causal associations for depression, schizophrenia, suicide ideation and bipolar disorder. Meanwhile, we found some novelty associations for insomnia, post-traumatic stress disorder and a possible association for anxiety. However, it should be cautious to interpret causal associations of smoking initiation with suicide attempts, schizophrenia, bipolar disorder and anxiety due to the limitation of instrumental variables used. More studies are needed to explore these association in a causal fashion.

A bidirectional association has been observed between smoking and several psychiatric disorders, such as bipolar disorder^36^, schizophrenia^43^ and anxiety^44^ in observational studies. It has further been shown that individuals with depressive symptoms or past major depressive disorder were less likely to quit smoking compared with smokers without depression^45,46^. Even though meta-analyses found no psychiatric side-effects, such as depression and bad mood, derived from smoking cessation^47,48^ among general smoking quitters, some studies have detected an increased depression risk among those who attempt to quit unsuccessfully^49^ and somatic adverse reactions among individuals with nicotine replacement therapy^50^. Thus, considering mutually detrimental influence between smoking and psychiatric diseases, difficulty of smoking cessation among certain populations and possible side-effects of smoking cessation therapies, reducing smoking initiation at the beginning step is an effective strategy for mental illness prevention. However, a recent study suggested the protective effect of smoking against depression^14^. In our study, we also observed a reverse positive association between depression and smoking initiation, which supported that patients with depressive symptoms were more likely to start cigarette smoking possibly due to a depression-releasing effect derived from smoking. Thus, whether stopping smoking should be recommended among patietns with depression needs to be further assessed.

Mechanisms explaining the comorbidity of smoking (nicotine dependence) with psychiatric diseases have been well acknowledged, especially for the prevalent smoking addiction among schizophrenia patients^13^. Experimental and genetic evidence shows that nicotine can normalize several deficits, such as attention deficits, among individuals with certain psychiatric problems via nicotinic acetylcholine receptors^13^. Antidepressant actions via monoamine oxidase inhibition (from unknown components from cigarettes) and nicotine-derived compensation effects of psychiatric medications also rationalize the phenomenon of more smokers among psychiatric patients compared with healthy adults^13^. Although the mechanisms are not clearly clarified about the increased risk of psychiatric diseases among smokers, pathways listed above may play a role. Another possible mechanism linking smoking and psychiatric disorders is the effects of nicotine on the dopamine system^3,51^. Nicotine has been also proposed to influence the dopamine system through induction of supersensitivity of D2 receptors, which shows associations with the risk of schizophrenia and other psychotic symptoms^3,52^. In addition, cigarette smoking has been suggested to increase the risk of anxiety by influencing neurotransmitter systems, the immune system, oxidative and nitrogen stress, mitochondrial function, and epigenetic regulation^53^. Given genetic and phenotypical overlapping^54–56^ across major psychiatric disorders, above smoking-casued anxiety-related pathways, may play roles in other specific mental diseases.

There are several advantages of our study. The MR approach minimized reverse causality, residual confounding, and misclassification, which potentially exist in observational studies. Population stratification bias was reduced in this study because SNP selection and summary-level data of psychiatric disorders were merely based on individuals of European descent and population structure was adjusted for in the GWASs. Moreover, the consistency in results across sensitivity analyses and no evidence of horizontal pleiotropy in the MR-Egger analysis indicate that our findings are unlikely biased by horizontal pleiotropy. However, our study has some limitations. The major limitation is the insufficient power to support a significant association between smoking initiation and anxiety, which needs to be verified in future studies. We had weak instrument bias or false causal direction in several analyses, which hindered the causal inference on these associations. In addition, because we only used the data from European-descent individuals, it should be cautious to generalize our findings to other populations.

In conclusion, this MR study provided evidence that smoking initiation is causally associated with an increased risk of a number of psychiatric disorders. However, whether smoking increases the risk of suicide attempts, schizophrenia, bipolar disorder and anxiety warrants future study. Reducing smoking initiation might be an effective strategy to prevent psychiatric disorders, but possibly not among patients with depression.

## Supplementary information

Supplementary Information
