# Supplementary material for: Associations of cigarette smoking with psychiatric disorders: evidence from a two-sample Mendelian randomization study
Source: Sci Rep. 2020 Aug 14;10:13807. doi: 10.1038/s41598-020-70458-4 (PMC7427799; doi:10.1038/s41598-020-70458-4)
Supplement: Supplementary file 1 — Supplementary Information [file 41598_2020_70458_MOESM1_ESM.docx]

**Supplements**

**Associations of cigarette smoking with psychiatric disorders: evidence from a two-sample Mendelian randomization study**

Shuai Yuan, Honghui Yao, Susanna C. Larsson

**Contents**

Supplementary Table 1. Detailed information of instrumental variables for smoking initiation

Supplementary Table 2. Definition of psychiatric disorders included in the present MR study

Supplementary Table 3. Heterogeneity in the association between smoking initiation and psychiatric disorders

Supplementary Table 4. The causal effect of depression on smoking initiation

**Supplementary Table 1.** Detailed information of instrumental variables for smoking initiation

| **rsID** | **Chr** | **Position** | **EA** | **NEA** | **Beta** | **SE** | **P** | **EAF** | **Nearby gene** | **N** | **Excluded ^a^** |
| --- | --- | --- | --- | --- | --- | --- | --- | --- | --- | --- | --- |
| rs12130857 | 1 | 7791461 | G | A | 0.018 | 0.003 | 3.65E-11 | 0.68 | *Intron:CAMTA1* | 1209400 |  |
| rs301807 | 1 | 8484823 | G | A | 0.018 | 0.003 | 2.5E-12 | 0.57 | *Intron:LOC102724552\|RERE* | 1225687 |  |
| rs3820277 | 1 | 18436657 | G | T | 0.019 | 0.003 | 1.57E-13 | 0.47 | *Intron:IGSF21* | 1198121 |  |
| rs1889571 | 1 | 32195819 | G | T | 0.022 | 0.004 | 4.19E-09 | 0.13 | *Intron:ADGRB2* | 1216367 |  |
| rs10914684 | 1 | 33795572 | G | A | 0.016 | 0.003 | 6.32E-09 | 0.68 | *Intron:PHC2* | 1218534 |  |
| rs2637869 | 1 | 38757237 | A | G | 0.018 | 0.003 | 6.54E-11 | 0.30 | *Intergenic* | 1217469 |  |
| rs12755632 | 1 | 41776623 | A | G | 0.015 | 0.003 | 1.93E-08 | 0.68 | *Intergenic* | 1221471 |  |
| rs951740 | 1 | 44011737 | A | G | 0.030 | 0.003 | 3.82E-29 | 0.63 | *Intron:PTPRF* | 1218849 |  |
| rs925524 | 1 | 46496709 | G | A | 0.016 | 0.003 | 2.94E-08 | 0.71 | *Synonymous:MAST2* | 1220767 |  |
| rs12022778 | 1 | 50603995 | C | A | 0.027 | 0.003 | 3.18E-17 | 0.20 | *Intron:ELAVL4* | 1223278 |  |
| rs11587399 | 1 | 50861071 | A | T | 0.018 | 0.003 | 7.25E-09 | 0.78 | *Intergenic* | 1216647 |  |
| rs4912332 | 1 | 58815243 | T | C | 0.014 | 0.003 | 2.94E-08 | 0.49 | *Intergenic* | 1222704 |  |
| rs1937443 | 1 | 66469643 | G | C | 0.020 | 0.003 | 1.79E-15 | 0.56 | *Intron:PDE4B* | 1220684 |  |
| rs1022528 | 1 | 71490122 | A | G | 0.017 | 0.003 | 8.48E-11 | 0.34 | *Intron:PTGER3* | 1219075 |  |
| rs12740789 | 1 | 72752073 | G | A | 0.028 | 0.003 | 1.18E-17 | 0.82 | *Intergenic* | 1224091 |  |
| rs80054503 | 1 | 72900406 | T | C | 0.024 | 0.004 | 3.1E-09 | 0.88 | *Intergenic* | 875823 | 1 |
| rs10789369 | 1 | 73824909 | A | G | 0.023 | 0.003 | 3.39E-19 | 0.39 | *Intergenic* | 1227940 |  |
| rs1514176 | 1 | 74991596 | G | A | 0.019 | 0.003 | 7.67E-14 | 0.42 | *Intron:FPGT-TNNI3K\|TNNI3K* | 1231299 |  |
| rs10873871 | 1 | 76689019 | G | A | 0.017 | 0.003 | 2.82E-08 | 0.21 | *Intron:ST6GALNAC3* | 1215942 |  |
| rs11162019 | 1 | 87913176 | C | T | 0.015 | 0.003 | 5.06E-09 | 0.64 | *Intergenic* | 1224661 |  |
| rs1008078 | 1 | 91189731 | T | C | 0.023 | 0.003 | 1.63E-18 | 0.40 | *Intergenic* | 1204464 |  |
| rs1935571 | 1 | 96414335 | T | G | 0.016 | 0.003 | 6.99E-10 | 0.52 | *Intergenic* | 1227897 |  |
| rs12027999 | 1 | 1.54E+08 | T | C | 0.024 | 0.004 | 5.33E-10 | 0.88 | *Intron:UBAP2L* | 1225575 |  |
| rs45444697 | 1 | 1.55E+08 | G | C | 0.020 | 0.003 | 2.72E-10 | 0.21 | *Intron:ADAM15\|DCST1-AS1* | 1191341 |  |
| rs2901785 | 1 | 1.74E+08 | G | A | 0.017 | 0.003 | 1.47E-11 | 0.55 | *Intron:LOC102724601* | 1229058 |  |
| rs147052174 | 1 | 1.8E+08 | T | G | 0.062 | 0.010 | 2.3E-10 | 0.02 | *Nonsynonymous:FAM163A* | 1151582 |  |
| rs35656245 | 1 | 1.91E+08 | A | G | 0.016 | 0.003 | 2.23E-08 | 0.28 | *Intergenic* | 1221162 |  |
| rs12739243 | 1 | 2.1E+08 | T | C | 0.021 | 0.003 | 4.45E-12 | 0.78 | *Intron:SYT14* | 1225617 |  |
| rs12563365 | 1 | 2.37E+08 | A | G | 0.017 | 0.003 | 1.05E-10 | 0.56 | *Intron:ACTN2* | 1218040 |  |
| rs876793 | 1 | 2.38E+08 | T | C | 0.018 | 0.003 | 5.69E-11 | 0.65 | *Intron:RYR2* | 1159331 |  |
| rs114976176 | 2 | 264621 | A | C | 0.016 | 0.003 | 6.04E-09 | 0.65 | *Intron:SH3YL1* | 1215932 |  |
| rs62106258 | 2 | 417167 | T | C | 0.045 | 0.006 | 3.33E-14 | 0.95 | *Intergenic* | 995317 |  |
| rs6731872 | 2 | 624205 | G | T | 0.032 | 0.003 | 5.35E-21 | 0.83 | *Intergenic* | 1221990 |  |
| rs1022376 | 2 | 22067213 | T | C | 0.015 | 0.003 | 1.66E-08 | 0.48 | *Intergenic* | 1158920 |  |
| rs61533748 | 2 | 22582968 | C | T | 0.017 | 0.003 | 2.82E-11 | 0.38 | *Intergenic* | 1214227 |  |
| rs72790288 | 2 | 29513404 | G | A | 0.046 | 0.008 | 3.28E-09 | 0.97 | *Intron:ALK* | 1150434 |  |
| rs2710634 | 2 | 32808804 | T | C | 0.018 | 0.003 | 3.36E-12 | 0.48 | *Intron:BIRC6* | 1210154 |  |
| rs62137126 | 2 | 44250149 | A | G | 0.024 | 0.004 | 1.31E-09 | 0.88 | *Intergenic* | 1203705 |  |
| rs1004787 | 2 | 45159091 | A | G | 0.028 | 0.003 | 1.11E-28 | 0.55 | *Intron:LINC01833* | 1214872 | 1 |
| rs7598402 | 2 | 50735943 | C | G | 0.015 | 0.003 | 7.38E-09 | 0.51 | *Intron:NRXN1* | 1217331 |  |
| rs10490159 | 2 | 51341259 | T | C | 0.017 | 0.003 | 3.86E-11 | 0.39 | *Intron:LOC730100* | 1213239 |  |
| rs1518393 | 2 | 58171220 | C | A | 0.017 | 0.003 | 1.3E-10 | 0.62 | *Intron:VRK2* | 1199847 | 1 |
| rs17616642 | 2 | 59022210 | A | G | 0.017 | 0.003 | 2.1E-08 | 0.75 | *Intron:LINC01122* | 1210471 |  |
| rs6730325 | 2 | 59315828 | G | A | 0.015 | 0.003 | 2.1E-08 | 0.39 | *Intergenic* | 1224503 |  |
| rs2539706 | 2 | 59819545 | A | G | 0.016 | 0.003 | 1.95E-10 | 0.53 | *Intergenic* | 1229329 |  |
| rs7585579 | 2 | 60024857 | G | C | 0.020 | 0.003 | 5.48E-15 | 0.50 | *Intergenic* | 1144119 |  |
| rs1863161 | 2 | 60139524 | A | G | 0.015 | 0.003 | 2.34E-09 | 0.56 | *Intergenic* | 1225789 |  |
| rs359247 | 2 | 60477052 | T | A | 0.022 | 0.003 | 9.89E-17 | 0.64 | *Intergenic* | 1226762 |  |
| rs62180324 | 2 | 63416606 | G | A | 0.020 | 0.003 | 3.91E-10 | 0.79 | *Intron:WDPCP* | 1226719 |  |
| rs6750107 | 2 | 80748807 | A | G | 0.015 | 0.003 | 2.6E-08 | 0.39 | *Intron:CTNNA2* | 1204380 |  |
| rs12714017 | 2 | 80999398 | C | T | 0.015 | 0.003 | 3.65E-09 | 0.51 | *Intergenic* | 1156271 |  |
| rs56208390 | 2 | 83247997 | G | A | 0.022 | 0.004 | 2.68E-08 | 0.12 | *Intergenic* | 1222928 |  |
| rs11692435 | 2 | 98275354 | A | G | 0.025 | 0.005 | 4.47E-08 | 0.08 | *Nonsynonymous:ACTR1B* | 1192616 |  |
| rs13392222 | 2 | 1.01E+08 | A | C | 0.023 | 0.004 | 1.93E-10 | 0.86 | *Intron:AFF3* | 1211975 |  |
| rs1901477 | 2 | 1.04E+08 | G | A | 0.030 | 0.003 | 2.07E-31 | 0.51 | *Intergenic* | 1173560 |  |
| rs11889814 | 2 | 1.04E+08 | A | C | 0.021 | 0.004 | 3.44E-08 | 0.87 | *Intergenic* | 1230166 |  |
| rs3811038 | 2 | 1.13E+08 | C | T | 0.019 | 0.003 | 1.58E-11 | 0.28 | *Intron:TTL* | 1222366 |  |
| rs75210106 | 2 | 1.13E+08 | C | T | 0.019 | 0.003 | 2.33E-08 | 0.82 | *Intron:TTL* | 1210760 |  |
| rs34399632 | 2 | 1.38E+08 | G | A | 0.019 | 0.003 | 1.46E-10 | 0.23 | *Intron:THSD7B* | 1219768 |  |
| rs74697736 | 2 | 1.45E+08 | A | G | 0.022 | 0.003 | 2.43E-15 | 0.29 | *Intergenic* | 1218275 |  |
| rs6756212 | 2 | 1.46E+08 | C | T | 0.034 | 0.003 | 3.49E-40 | 0.47 | *Intergenic* | 1221091 |  |
| rs3076896 | 2 | 1.46E+08 | A | G | 0.023 | 0.003 | 1.99E-16 | 0.39 | *Intergenic* | 853934 | 1 |
| rs16826827 | 2 | 1.48E+08 | T | C | 0.022 | 0.004 | 9.17E-09 | 0.88 | *Intergenic* | 1219788 |  |
| rs1445649 | 2 | 1.56E+08 | C | T | 0.021 | 0.003 | 8.48E-16 | 0.54 | *Intron:KCNJ3* | 1228573 |  |
| rs1722666 | 2 | 1.62E+08 | T | C | 0.016 | 0.003 | 2.17E-08 | 0.73 | *Intergenic* | 1224456 |  |
| rs11678980 | 2 | 1.62E+08 | A | G | 0.018 | 0.003 | 5.19E-12 | 0.45 | *Exon:LINC01806* | 1125962 |  |
| rs12474587 | 2 | 1.63E+08 | T | G | 0.024 | 0.003 | 4.83E-21 | 0.43 | *Intron:SLC4A10* | 1219624 |  |
| rs357304 | 2 | 1.65E+08 | C | T | 0.017 | 0.003 | 5.4E-09 | 0.73 | *Intergenic* | 1204924 |  |
| rs13007361 | 2 | 1.66E+08 | A | G | 0.018 | 0.003 | 2.29E-08 | 0.21 | *Intergenic* | 1216581 |  |
| rs7600835 | 2 | 1.73E+08 | G | A | 0.015 | 0.003 | 1.8E-08 | 0.66 | *Intergenic* | 1185909 |  |
| rs6750529 | 2 | 1.82E+08 | T | C | 0.020 | 0.003 | 9.26E-12 | 0.74 | *Intron:LINC01934* | 1222047 |  |
| rs17229285 | 2 | 2E+08 | C | T | 0.015 | 0.003 | 1.27E-09 | 0.50 | *Intergenic* | 1222863 |  |
| rs3115418 | 2 | 2.01E+08 | T | C | 0.014 | 0.003 | 2.79E-08 | 0.55 | *Intergenic* | 1229023 |  |
| rs62193862 | 2 | 2.03E+08 | A | G | 0.024 | 0.004 | 1.99E-08 | 0.10 | *Intergenic* | 1172456 |  |
| rs4674916 | 2 | 2.25E+08 | C | A | 0.018 | 0.003 | 3.06E-11 | 0.67 | *Intron:CUL3* | 1230534 |  |
| rs4674993 | 2 | 2.26E+08 | A | G | 0.024 | 0.003 | 4.85E-14 | 0.80 | *Intron:NYAP2* | 1224561 |  |
| rs11713899 | 3 | 2365026 | C | A | 0.019 | 0.003 | 3.15E-08 | 0.17 | *Intron:CNTN4* | 1216134 |  |
| rs748832 | 3 | 16851202 | G | A | 0.017 | 0.003 | 6.6E-11 | 0.37 | *Intergenic* | 1230928 |  |
| rs10446419 | 3 | 25725501 | A | G | 0.020 | 0.003 | 5.05E-10 | 0.79 | *Intergenic* | 1130264 |  |
| rs13319205 | 3 | 47800216 | A | T | 0.017 | 0.003 | 3.77E-09 | 0.29 | *Intron:SMARCC1* | 1196585 |  |
| rs3172494 | 3 | 48731487 | G | T | 0.029 | 0.004 | 3.4E-13 | 0.89 | *Utr3:IP6K2* | 1212359 |  |
| rs2526390 | 3 | 50192760 | T | C | 0.020 | 0.003 | 3.62E-14 | 0.33 | *Intron:SEMA3F\|SEMA3F-AS1* | 1216116 |  |
| rs2276825 | 3 | 52886605 | C | T | 0.019 | 0.003 | 1.89E-10 | 0.25 | *Intron:STIMATE\|TMEM110-MUSTN1* | 1224287 |  |
| rs2306866 | 3 | 53766212 | A | T | 0.017 | 0.003 | 1.89E-10 | 0.39 | *Intron:CACNA1D* | 1215989 |  |
| rs73831818 | 3 | 55988394 | G | A | 0.032 | 0.005 | 5.46E-09 | 0.06 | *Intron:ERC2* | 1227986 |  |
| rs1910236 | 3 | 59434420 | A | G | 0.015 | 0.003 | 9.91E-09 | 0.47 | *Intergenic* | 1219509 |  |
| rs7640107 | 3 | 59966156 | C | T | 0.014 | 0.003 | 3.46E-08 | 0.57 | *Intron:FHIT* | 1226138 |  |
| rs2734390 | 3 | 60459291 | G | A | 0.015 | 0.003 | 2.09E-08 | 0.37 | *Intron:FHIT* | 1218951 |  |
| rs221988 | 3 | 64234307 | A | C | 0.015 | 0.003 | 1.43E-08 | 0.62 | *Intergenic* | 1192013 |  |
| rs2196356 | 3 | 70890288 | G | C | 0.019 | 0.003 | 2.45E-11 | 0.71 | *Intergenic* | 1223929 | 1 |
| rs11128203 | 3 | 71064431 | A | T | 0.020 | 0.003 | 1.29E-15 | 0.53 | *Intron:FOXP1* | 1209197 |  |
| rs62246017 | 3 | 71483084 | G | A | 0.016 | 0.003 | 3.03E-09 | 0.68 | *Intron:FOXP1* | 1184766 |  |
| rs4543050 | 3 | 74954560 | T | A | 0.022 | 0.003 | 1.45E-11 | 0.82 | *Intergenic* | 1202063 |  |
| rs6782116 | 3 | 77176032 | C | T | 0.015 | 0.003 | 1.46E-08 | 0.59 | *Intron:ROBO2* | 1159443 |  |
| rs13066050 | 3 | 81325861 | T | C | 0.019 | 0.003 | 1.93E-09 | 0.21 | *Intergenic* | 1225588 |  |
| rs12633090 | 3 | 83241365 | G | C | 0.023 | 0.003 | 3.16E-12 | 0.82 | *Intergenic* | 1205358 |  |
| rs1549979 | 3 | 85460131 | C | T | 0.025 | 0.003 | 8.8E-21 | 0.39 | *Intron:CADM2* | 1222403 |  |
| rs74664784 | 3 | 85475292 | T | C | 0.020 | 0.003 | 9.34E-13 | 0.62 | *Intron:CADM2* | 974268 | 1 |
| rs57153235 | 3 | 85902536 | T | G | 0.019 | 0.003 | 1.56E-12 | 0.68 | *Intron:CADM2* | 1215104 |  |
| rs6437769 | 3 | 1.08E+08 | T | C | 0.014 | 0.003 | 3.74E-08 | 0.58 | *Intergenic* | 1228839 |  |
| rs9288999 | 3 | 1.14E+08 | A | G | 0.017 | 0.003 | 1.5E-09 | 0.74 | *Intron:ZBTB20* | 1202717 |  |
| rs6438436 | 3 | 1.18E+08 | T | C | 0.025 | 0.003 | 5.33E-14 | 0.82 | *Intergenic* | 1191951 |  |
| rs12053870 | 3 | 1.18E+08 | G | T | 0.016 | 0.003 | 1.02E-09 | 0.54 | *Intron:LOC105374060* | 1208216 |  |
| rs9826984 | 3 | 1.32E+08 | G | A | 0.014 | 0.003 | 3.87E-08 | 0.46 | *Intergenic* | 1221863 |  |
| rs2279829 | 3 | 1.47E+08 | C | T | 0.017 | 0.003 | 2.05E-08 | 0.78 | *Utr3:ZIC4* | 1222607 |  |
| rs2319545 | 3 | 1.48E+08 | A | C | 0.023 | 0.004 | 8.3E-11 | 0.15 | *Intergenic* | 1202391 |  |
| rs10935779 | 3 | 1.5E+08 | C | T | 0.014 | 0.003 | 2.95E-08 | 0.59 | *Intron:RNF13* | 1223044 |  |
| rs963354 | 3 | 1.57E+08 | A | C | 0.015 | 0.003 | 4.21E-08 | 0.69 | *Intergenic* | 1224961 |  |
| rs1714521 | 3 | 1.58E+08 | A | C | 0.016 | 0.003 | 3.07E-10 | 0.59 | *Intron:LOC100996447* | 1223591 |  |
| rs1449012 | 3 | 1.59E+08 | C | T | 0.015 | 0.003 | 1.77E-09 | 0.54 | *Intron:IQCJ-SCHIP1\|SCHIP1* | 1191572 |  |
| rs9850597 | 3 | 1.62E+08 | G | A | 0.019 | 0.003 | 1.65E-08 | 0.18 | *Intergenic* | 1222336 |  |
| rs1187820 | 3 | 1.73E+08 | C | T | 0.014 | 0.003 | 2.69E-08 | 0.56 | *Intergenic* | 1148531 |  |
| rs16828799 | 3 | 1.73E+08 | T | G | 0.020 | 0.004 | 1.83E-08 | 0.16 | *Intron:NLGN1* | 1220267 |  |
| rs9841807 | 3 | 1.76E+08 | T | C | 0.016 | 0.003 | 1.35E-08 | 0.27 | *Intergenic* | 1228503 |  |
| rs7631379 | 3 | 1.81E+08 | C | T | 0.021 | 0.003 | 3.94E-11 | 0.21 | *Intron:SOX2-OT* | 1138636 |  |
| rs4140932 | 4 | 15458598 | T | A | 0.014 | 0.003 | 4.89E-08 | 0.57 | *Intergenic* | 1222445 |  |
| rs12642744 | 4 | 28027176 | G | T | 0.017 | 0.003 | 2.82E-08 | 0.26 | *Intergenic* | 1173199 |  |
| rs59537158 | 4 | 28246049 | T | C | 0.022 | 0.003 | 4.62E-13 | 0.21 | *Intergenic* | 1137994 |  |
| rs1389171 | 4 | 28822284 | T | A | 0.017 | 0.003 | 4.45E-09 | 0.76 | *Intergenic* | 1220803 |  |
| rs55944129 | 4 | 29082156 | T | C | 0.018 | 0.003 | 1.06E-09 | 0.73 | *Intergenic* | 1224851 |  |
| rs58400863 | 4 | 31184484 | G | A | 0.020 | 0.003 | 4.89E-14 | 0.65 | *Intron:LINC02497* | 1198795 |  |
| rs7657022 | 4 | 35501032 | G | A | 0.018 | 0.003 | 7.34E-13 | 0.49 | *Intergenic* | 1229419 |  |
| rs55900829 | 4 | 35514712 | T | A | 0.019 | 0.003 | 5.63E-12 | 0.33 | *Intergenic* | 943683 | 1 |
| rs112725451 | 4 | 68017710 | T | C | 0.026 | 0.003 | 1.65E-14 | 0.17 | *Intergenic* | 1227231 |  |
| rs1160685 | 4 | 94052854 | G | C | 0.015 | 0.003 | 2.31E-09 | 0.45 | *Intron:GRID2* | 1227684 |  |
| rs1435479 | 4 | 94550450 | T | G | 0.016 | 0.003 | 5.68E-09 | 0.29 | *Intron:GRID2* | 1226852 |  |
| rs3934797 | 4 | 1.12E+08 | G | A | 0.021 | 0.003 | 1.12E-10 | 0.82 | *Intergenic* | 1205016 |  |
| rs71602617 | 4 | 1.36E+08 | C | T | 0.018 | 0.003 | 2.1E-08 | 0.78 | *Intergenic* | 1147175 |  |
| rs7696257 | 4 | 1.37E+08 | A | G | 0.015 | 0.003 | 6.78E-09 | 0.37 | *Intergenic* | 1206055 |  |
| rs13109980 | 4 | 1.41E+08 | G | A | 0.022 | 0.003 | 3.37E-16 | 0.67 | *Intron:MAML3* | 1218048 |  |
| rs1116690 | 4 | 1.44E+08 | G | A | 0.016 | 0.003 | 2.16E-08 | 0.74 | *Intron:INPP4B* | 1228175 |  |
| rs13110073 | 4 | 1.48E+08 | T | C | 0.025 | 0.003 | 3.24E-21 | 0.61 | *Intron:TTC29* | 1228665 |  |
| rs28717373 | 4 | 1.48E+08 | C | T | 0.016 | 0.003 | 6.16E-10 | 0.64 | *Intergenic* | 1215277 | 1 |
| rs62340589 | 4 | 1.77E+08 | C | G | 0.017 | 0.003 | 4.31E-08 | 0.20 | *Intron:GPM6A* | 1210810 |  |
| rs12517438 | 5 | 30842054 | G | T | 0.015 | 0.003 | 1.89E-09 | 0.54 | *Intergenic* | 1224523 |  |
| rs35375873 | 5 | 43190647 | G | C | 0.027 | 0.004 | 3.29E-11 | 0.89 | *Intergenic* | 1038425 |  |
| rs986714 | 5 | 50821338 | A | T | 0.016 | 0.003 | 4.13E-10 | 0.56 | *Intergenic* | 1225436 |  |
| rs71592686 | 5 | 60121271 | C | T | 0.021 | 0.003 | 3.85E-13 | 0.27 | *Intron:ELOVL7* | 1221100 |  |
| rs2028269 | 5 | 79308315 | A | G | 0.016 | 0.003 | 5.19E-10 | 0.40 | *Intron:THBS4* | 1225374 |  |
| rs6874731 | 5 | 80263865 | G | T | 0.015 | 0.003 | 1.83E-09 | 0.48 | *Intron:RASGRF2* | 1218833 |  |
| rs6452785 | 5 | 87685500 | C | T | 0.027 | 0.003 | 4.69E-26 | 0.53 | *Intron:TMEM161B-AS1* | 1225500 |  |
| rs10805858 | 5 | 88873832 | T | A | 0.018 | 0.003 | 1.88E-11 | 0.34 | *Intergenic* | 1209377 |  |
| rs181508347 | 5 | 91366274 | G | T | 0.081 | 0.013 | 4.95E-10 | 0.01 | *Intergenic* | 1148155 | 1 |
| rs42417 | 5 | 94198290 | T | C | 0.017 | 0.003 | 8.27E-10 | 0.69 | *Intron:MCTP1* | 1210401 |  |
| rs72780746 | 5 | 1.04E+08 | T | C | 0.026 | 0.003 | 2.05E-14 | 0.83 | *Intergenic* | 1222477 |  |
| rs10060196 | 5 | 1.06E+08 | A | C | 0.018 | 0.003 | 1.29E-12 | 0.58 | *Intergenic* | 1221528 |  |
| rs72789626 | 5 | 1.07E+08 | T | A | 0.026 | 0.004 | 5.13E-12 | 0.86 | *Intron:EFNA5* | 1216167 |  |
| rs17165769 | 5 | 1.07E+08 | G | A | 0.016 | 0.003 | 9.56E-10 | 0.39 | *Intron:FBXL17* | 1226874 |  |
| rs329124 | 5 | 1.34E+08 | A | G | 0.016 | 0.003 | 1.96E-10 | 0.57 | *Intron:JADE2* | 1224057 |  |
| rs1385108 | 5 | 1.55E+08 | T | C | 0.019 | 0.003 | 3.84E-10 | 0.24 | *Intergenic* | 1220995 |  |
| rs1173461 | 5 | 1.58E+08 | T | C | 0.017 | 0.003 | 9.51E-10 | 0.33 | *Intergenic* | 1217455 |  |
| rs11956866 | 5 | 1.61E+08 | T | G | 0.015 | 0.003 | 7.82E-09 | 0.43 | *Intergenic* | 1227737 |  |
| rs3909281 | 5 | 1.65E+08 | G | T | 0.021 | 0.003 | 1.62E-16 | 0.54 | *Intergenic* | 1214873 |  |
| rs3843905 | 5 | 1.65E+08 | C | T | 0.015 | 0.003 | 5.41E-09 | 0.60 | *Intergenic* | 1221926 |  |
| rs79476395 | 5 | 1.66E+08 | G | A | 0.033 | 0.005 | 1.04E-11 | 0.07 | *Intergenic* | 1197139 |  |
| rs6890961 | 5 | 1.67E+08 | C | T | 0.019 | 0.003 | 2.13E-13 | 0.38 | *Intron:TENM2* | 1208641 |  |
| rs4044321 | 5 | 1.67E+08 | A | G | 0.023 | 0.003 | 1.75E-17 | 0.36 | *Intron:TENM2* | 1226412 |  |
| rs2173019 | 5 | 1.68E+08 | A | T | 0.028 | 0.003 | 2.98E-17 | 0.18 | *Intron:TENM2* | 1217617 |  |
| rs10042827 | 5 | 1.7E+08 | C | T | 0.017 | 0.003 | 9.41E-10 | 0.68 | *Intron:RANBP17* | 1218220 |  |
| rs359431 | 5 | 1.73E+08 | C | T | 0.014 | 0.003 | 3.16E-08 | 0.44 | *Intergenic* | 1227870 |  |
| rs1059490 | 6 | 26171250 | T | C | 0.019 | 0.003 | 2.16E-12 | 0.63 | *Utr3:HIST1H2BD* | 1217552 |  |
| rs6932350 | 6 | 26571629 | A | T | 0.015 | 0.003 | 5.13E-09 | 0.45 | *Intron:LOC105374988* | 1209778 | 1 |
| rs1150668 | 6 | 28129789 | T | G | 0.019 | 0.003 | 8.54E-13 | 0.58 | *Intron:ZNF192P1* | 1208391 |  |
| rs1632941 | 6 | 29796685 | T | C | 0.016 | 0.003 | 6.67E-10 | 0.54 | *Intron:HLA-G* | 1187767 |  |
| rs3218116 | 6 | 41901763 | C | T | 0.020 | 0.003 | 1.05E-11 | 0.74 | *Intergenic* | 1222087 |  |
| rs160631 | 6 | 52895230 | T | G | 0.017 | 0.003 | 1.87E-09 | 0.27 | *Intron:ICK* | 1229351 |  |
| rs7743165 | 6 | 67521222 | G | T | 0.019 | 0.003 | 4.15E-14 | 0.50 | *Intergenic* | 1227607 |  |
| rs79180767 | 6 | 67540984 | T | C | 0.020 | 0.003 | 7E-12 | 0.25 | *Intergenic* | 944917 | 1 |
| rs10945141 | 6 | 69470709 | A | G | 0.018 | 0.003 | 3.59E-10 | 0.26 | *Intron:ADGRB3* | 1222190 |  |
| rs17554906 | 6 | 92226609 | C | G | 0.014 | 0.003 | 3.14E-08 | 0.44 | *Intergenic* | 1223182 |  |
| rs619087 | 6 | 94175279 | G | A | 0.014 | 0.003 | 3.1E-08 | 0.42 | *Intergenic* | 1226268 |  |
| rs6568832 | 6 | 97702876 | A | G | 0.019 | 0.003 | 1.74E-10 | 0.75 | *Intron:MIR548H3\|MMS22L* | 1226188 |  |
| rs12195240 | 6 | 98636905 | A | G | 0.025 | 0.003 | 1.08E-18 | 0.29 | *Intergenic* | 1226630 |  |
| rs6936160 | 6 | 1E+08 | T | C | 0.020 | 0.003 | 4.2E-13 | 0.70 | *Intergenic* | 1222678 |  |
| rs12530388 | 6 | 1.01E+08 | A | C | 0.018 | 0.003 | 5.83E-13 | 0.49 | *Utr5:ASCC3* | 1225293 |  |
| rs3800227 | 6 | 1.09E+08 | G | A | 0.017 | 0.003 | 3.64E-09 | 0.74 | *Intron:FOXO3* | 1221922 |  |
| rs118202 | 6 | 1.12E+08 | G | T | 0.037 | 0.003 | 1.9E-29 | 0.19 | *Intron:REV3L* | 1227421 |  |
| rs73008357 | 6 | 1.56E+08 | A | C | 0.022 | 0.004 | 2.44E-08 | 0.88 | *Intergenic* | 1164746 |  |
| rs9331343 | 6 | 1.58E+08 | T | C | 0.014 | 0.003 | 3.9E-08 | 0.43 | *Intron:TMEM242* | 1148931 |  |
| rs10698713 | 6 | 1.59E+08 | G | A | 0.034 | 0.006 | 2.38E-09 | 0.95 | *Intron:TULP4* | 1196529 | 1 |
| rs1737329 | 6 | 1.64E+08 | G | C | 0.017 | 0.003 | 5.08E-09 | 0.74 | *Intergenic* | 1223438 |  |
| rs10272990 | 7 | 1703675 | T | C | 0.021 | 0.003 | 1.27E-14 | 0.67 | *Intergenic* | 1179522 |  |
| rs6948707 | 7 | 1870794 | G | T | 0.024 | 0.003 | 4.24E-21 | 0.42 | *Intron:MAD1L1* | 1216075 |  |
| rs10259715 | 7 | 3329967 | T | A | 0.019 | 0.003 | 6.42E-09 | 0.79 | *Intergenic* | 940717 | 1 |
| rs13237637 | 7 | 3503207 | G | C | 0.024 | 0.003 | 1.54E-20 | 0.52 | *Intron:SDK1* | 1221334 |  |
| rs79631993 | 7 | 69432311 | A | C | 0.017 | 0.003 | 3.67E-08 | 0.78 | *Intron:AUTS2* | 1028629 | 1 |
| rs7809303 | 7 | 69484366 | G | A | 0.021 | 0.003 | 3.48E-15 | 0.68 | *Intron:AUTS2* | 1230122 |  |
| rs7802996 | 7 | 77771983 | C | T | 0.021 | 0.003 | 1.06E-09 | 0.83 | *Intron:MAGI2* | 1227436 |  |
| rs1030015 | 7 | 78139581 | T | G | 0.014 | 0.003 | 2.15E-08 | 0.52 | *Intron:MAGI2* | 1229621 |  |
| rs4727189 | 7 | 88442568 | C | T | 0.015 | 0.003 | 3E-08 | 0.34 | *Intron:ZNF804B* | 1230628 |  |
| rs76841737 | 7 | 91281409 | C | G | 0.023 | 0.004 | 3.26E-08 | 0.90 | *Intergenic* | 1175208 |  |
| rs11768481 | 7 | 96629103 | C | A | 0.019 | 0.003 | 5.23E-12 | 0.66 | *Intron:DLX6-AS1* | 1163631 |  |
| rs1799068 | 7 | 97707069 | T | G | 0.017 | 0.003 | 2.59E-10 | 0.38 | *Intergenic* | 1223898 |  |
| rs13437771 | 7 | 99071478 | A | G | 0.027 | 0.004 | 1.39E-14 | 0.85 | *Intron:ZNF789* | 1216314 |  |
| rs11766326 | 7 | 1.11E+08 | T | C | 0.018 | 0.003 | 1.79E-11 | 0.49 | *Intron:IMMP2L* | 1164002 |  |
| rs6968380 | 7 | 1.15E+08 | G | A | 0.023 | 0.003 | 1.05E-17 | 0.32 | *Intergenic* | 1215518 |  |
| rs112913817 | 7 | 1.15E+08 | G | A | 0.078 | 0.012 | 9.28E-11 | 0.01 | *Intergenic* | 1066421 | 1 |
| rs10233018 | 7 | 1.18E+08 | G | A | 0.025 | 0.003 | 4.77E-22 | 0.52 | *Intergenic* | 1222792 |  |
| rs10953957 | 7 | 1.22E+08 | A | G | 0.014 | 0.003 | 3.66E-08 | 0.39 | *Intergenic* | 1196710 |  |
| rs77283305 | 7 | 1.33E+08 | G | A | 0.015 | 0.003 | 3.91E-08 | 0.69 | *Intron:CHCHD3* | 1221501 |  |
| rs10279261 | 7 | 1.34E+08 | G | A | 0.019 | 0.003 | 6.05E-13 | 0.38 | *Intron:EXOC4* | 1216245 |  |
| rs1561112 | 7 | 1.34E+08 | T | C | 0.015 | 0.003 | 3.84E-09 | 0.59 | *Intron:LRGUK* | 1215119 |  |
| rs2952251 | 8 | 10143164 | G | A | 0.016 | 0.003 | 4.24E-08 | 0.74 | *Intron:MSRA* | 1150969 |  |
| rs4326350 | 8 | 10763655 | C | G | 0.018 | 0.003 | 5.16E-12 | 0.51 | *Intron:XKR6* | 1223059 |  |
| rs11780471 | 8 | 27344719 | G | A | 0.039 | 0.005 | 1.57E-13 | 0.94 | *Intergenic* | 1211966 | 1 |
| rs11783093 | 8 | 27425349 | C | T | 0.047 | 0.003 | 2.07E-41 | 0.84 | *Intergenic* | 1214713 |  |
| rs1565735 | 8 | 27426077 | T | A | 0.019 | 0.003 | 1.33E-09 | 0.80 | *Intergenic* | 1214641 | 1 |
| rs7836565 | 8 | 52569449 | C | T | 0.016 | 0.003 | 4.36E-08 | 0.28 | *Intron:PXDNL* | 1229741 |  |
| rs13261666 | 8 | 59814666 | G | T | 0.020 | 0.003 | 4.36E-15 | 0.48 | *Intron:TOX* | 1226498 |  |
| rs3850736 | 8 | 64912021 | G | C | 0.019 | 0.003 | 6.43E-14 | 0.47 | *Intron:LOC102724623* | 1222296 |  |
| rs2063976 | 8 | 91096366 | C | T | 0.020 | 0.003 | 7.45E-14 | 0.34 | *Intergenic* | 1225654 |  |
| rs6993429 | 8 | 92733282 | C | A | 0.019 | 0.003 | 9.87E-14 | 0.55 | *Intergenic* | 1225057 |  |
| rs6986430 | 8 | 93048104 | T | C | 0.024 | 0.003 | 1.99E-15 | 0.78 | *Intron:RUNX1T1* | 1219933 |  |
| rs9987376 | 8 | 93190014 | T | G | 0.020 | 0.003 | 2.01E-15 | 0.43 | *Intergenic* | 1222251 |  |
| rs290601 | 8 | 1.15E+08 | T | C | 0.016 | 0.003 | 1.14E-08 | 0.27 | *Intergenic* | 1226515 |  |
| rs3847244 | 9 | 3025368 | T | C | 0.019 | 0.003 | 2.6E-13 | 0.47 | *Intergenic* | 1171615 |  |
| rs11791671 | 9 | 3398679 | T | C | 0.028 | 0.005 | 4.24E-08 | 0.07 | *Intron:RFX3* | 1214866 |  |
| rs7024924 | 9 | 8282399 | C | T | 0.019 | 0.003 | 1.9E-08 | 0.17 | *Intergenic* | 1229018 |  |
| rs6474609 | 9 | 10981069 | T | A | 0.016 | 0.003 | 1.71E-09 | 0.41 | *Intergenic* | 1211993 |  |
| rs1931431 | 9 | 11161799 | C | G | 0.018 | 0.003 | 8.56E-13 | 0.48 | *Intergenic* | 1221764 |  |
| rs7867822 | 9 | 20676454 | A | G | 0.015 | 0.003 | 2.76E-08 | 0.33 | *Intron:FOCAD* | 1228618 |  |
| rs10966092 | 9 | 23831658 | T | C | 0.020 | 0.003 | 1.12E-12 | 0.73 | *Intron:ELAVL2* | 1219742 |  |
| rs10969352 | 9 | 29747488 | A | T | 0.014 | 0.003 | 1.82E-08 | 0.50 | *Intergenic* | 1227461 |  |
| rs4877285 | 9 | 81354129 | G | A | 0.018 | 0.003 | 2.1E-11 | 0.33 | *Intergenic* | 1197842 |  |
| rs1930371 | 9 | 81444104 | C | T | 0.017 | 0.003 | 7.09E-09 | 0.76 | *Intergenic* | 1217523 |  |
| rs2378662 | 9 | 86707289 | A | G | 0.015 | 0.003 | 2.67E-09 | 0.54 | *Intron:LOC101927575* | 1213973 |  |
| rs1927901 | 9 | 1.21E+08 | T | C | 0.014 | 0.003 | 3.1E-08 | 0.45 | *Intergenic* | 1219221 |  |
| rs4837631 | 9 | 1.22E+08 | C | T | 0.015 | 0.003 | 2.03E-09 | 0.55 | *Intron:BRINP1* | 1227341 |  |
| rs1759433 | 9 | 1.28E+08 | A | G | 0.015 | 0.003 | 1.69E-09 | 0.48 | *Intron:GAPVD1* | 1227776 |  |
| rs34553878 | 9 | 1.34E+08 | G | A | 0.025 | 0.004 | 1.17E-09 | 0.11 | *Nonsynonymous:PRRC2B* | 1191115 |  |
| rs7026534 | 9 | 1.35E+08 | T | G | 0.017 | 0.003 | 2.68E-09 | 0.30 | *Intron:MED27* | 1229340 |  |
| rs10858334 | 9 | 1.38E+08 | G | C | 0.023 | 0.004 | 1.18E-09 | 0.14 | *Utr3:OLFM1* | 1119480 |  |
| rs10905461 | 10 | 8803551 | T | C | 0.016 | 0.003 | 2.36E-08 | 0.25 | *Intergenic* | 1227076 |  |
| rs7920501 | 10 | 10043159 | T | A | 0.016 | 0.003 | 1.25E-09 | 0.54 | *Intergenic* | 1225808 |  |
| rs1291821 | 10 | 11133823 | G | A | 0.014 | 0.003 | 1.39E-08 | 0.53 | *Intron:CELF2\|CELF2-AS2* | 1220680 |  |
| rs11258417 | 10 | 13533053 | C | T | 0.015 | 0.003 | 2.71E-08 | 0.61 | *Intron:BEND7* | 1229820 |  |
| rs7072776 | 10 | 22032942 | A | G | 0.022 | 0.003 | 5.66E-15 | 0.29 | *Intergenic* | 1223238 |  |
| rs2796793 | 10 | 36634124 | A | G | 0.014 | 0.003 | 1.55E-08 | 0.45 | *Intergenic* | 1226515 |  |
| rs1733760 | 10 | 56698174 | C | T | 0.015 | 0.003 | 6.7E-09 | 0.51 | *Intron:PCDH15* | 1223062 |  |
| rs7921378 | 10 | 63674885 | G | C | 0.023 | 0.003 | 6.1E-20 | 0.52 | *Intron:ARID5B* | 1216135 |  |
| rs7901883 | 10 | 1.03E+08 | G | A | 0.019 | 0.003 | 1.98E-10 | 0.77 | *Intron:BTRC* | 1216296 |  |
| rs11594623 | 10 | 1.04E+08 | C | T | 0.027 | 0.003 | 7.45E-20 | 0.23 | *Intergenic* | 1215577 |  |
| rs11191269 | 10 | 1.04E+08 | G | C | 0.018 | 0.003 | 4.61E-08 | 0.19 | *Intron:GBF1* | 1222474 |  |
| rs28408682 | 10 | 1.04E+08 | G | A | 0.017 | 0.003 | 1.41E-10 | 0.60 | *Intergenic* | 1224786 |  |
| rs12244388 | 10 | 1.05E+08 | A | G | 0.026 | 0.003 | 4.31E-22 | 0.35 | *Intron:AS3MT\|BORCS7-ASMT* | 1228272 |  |
| rs111842178 | 10 | 1.05E+08 | G | A | 0.022 | 0.003 | 2.24E-12 | 0.23 | *Intron:NT5C2* | 804613 | 1 |
| rs34970111 | 10 | 1.06E+08 | C | T | 0.015 | 0.003 | 1.28E-08 | 0.54 | *Intron:ITPRIP* | 1161167 |  |
| rs9787523 | 10 | 1.06E+08 | T | C | 0.016 | 0.003 | 1.42E-09 | 0.58 | *Intron:SORCS3* | 1206029 |  |
| rs11192347 | 10 | 1.07E+08 | G | A | 0.026 | 0.004 | 6.15E-10 | 0.90 | *Intron:SORCS3* | 1170998 |  |
| rs10885480 | 10 | 1.15E+08 | T | C | 0.019 | 0.003 | 3.83E-11 | 0.72 | *Intron:NRAP* | 1204794 |  |
| rs4752018 | 10 | 1.19E+08 | A | C | 0.019 | 0.003 | 4.42E-10 | 0.23 | *Intron:SHTN1* | 1223630 |  |
| rs9423279 | 10 | 1.26E+08 | C | G | 0.019 | 0.003 | 3.06E-12 | 0.36 | *Intergenic* | 1072640 |  |
| rs6265 | 11 | 27679916 | C | T | 0.029 | 0.003 | 2.81E-19 | 0.81 | *Nonsynonymous:BDNF* | 1220588 |  |
| rs4275621 | 11 | 28652996 | A | G | 0.021 | 0.003 | 3.76E-16 | 0.62 | *Intergenic* | 1218962 |  |
| rs62618693 | 11 | 32956492 | C | T | 0.035 | 0.006 | 2.09E-08 | 0.96 | *Nonsynonymous:QSER1* | 1133972 |  |
| rs2939756 | 11 | 41436297 | G | A | 0.016 | 0.003 | 7.45E-10 | 0.52 | *Intron:LRRC4C* | 1220880 |  |
| rs1381775 | 11 | 42442826 | T | C | 0.016 | 0.003 | 2.79E-08 | 0.29 | *Intergenic* | 1217378 |  |
| rs2959084 | 11 | 46078656 | A | G | 0.017 | 0.003 | 9.82E-10 | 0.70 | *Intron:PHF21A* | 1226775 |  |
| rs3740977 | 11 | 46393574 | C | T | 0.019 | 0.003 | 1.17E-08 | 0.17 | *Intron:DGKZ* | 1217443 |  |
| rs61886926 | 11 | 64133552 | C | T | 0.018 | 0.003 | 7.3E-12 | 0.62 | *Intron:RPS6KA4* | 1228233 |  |
| rs61884449 | 11 | 64485193 | T | C | 0.020 | 0.004 | 2.32E-08 | 0.15 | *Intron:NRXN2* | 1212196 |  |
| rs644740 | 11 | 65561468 | C | T | 0.014 | 0.003 | 3.67E-08 | 0.54 | *Intron:OVOL1* | 1224047 |  |
| rs7943721 | 11 | 73309393 | G | A | 0.021 | 0.003 | 3.58E-10 | 0.17 | *Intergenic* | 1204494 |  |
| rs7929518 | 11 | 85980958 | G | A | 0.019 | 0.003 | 2.55E-10 | 0.77 | *Intron:EED* | 1226211 |  |
| rs586699 | 11 | 92289734 | G | A | 0.015 | 0.003 | 7.29E-09 | 0.46 | *Intron:FAT3* | 1229566 |  |
| rs76460663 | 11 | 1.12E+08 | C | G | 0.042 | 0.006 | 4.15E-11 | 0.96 | *Intergenic* | 975306 |  |
| rs2155646 | 11 | 1.13E+08 | C | T | 0.038 | 0.003 | 9.44E-48 | 0.40 | *Intron:NCAM1* | 1228636 |  |
| rs78239456 | 11 | 1.13E+08 | A | T | 0.018 | 0.003 | 9.37E-12 | 0.62 | *Intron:NCAM1* | 998827 | 1 |
| rs1713676 | 11 | 1.14E+08 | A | G | 0.017 | 0.003 | 5.38E-11 | 0.48 | *Intergenic* | 1222330 |  |
| rs238896 | 11 | 1.14E+08 | G | A | 0.017 | 0.003 | 3.65E-11 | 0.51 | *Intron:ZBTB16* | 1227157 |  |
| rs540860 | 11 | 1.22E+08 | G | A | 0.018 | 0.003 | 5.75E-12 | 0.54 | *Intergenic* | 1220773 |  |
| rs1944689 | 11 | 1.22E+08 | T | G | 0.018 | 0.003 | 1.27E-08 | 0.79 | *Intergenic* | 1227799 |  |
| rs1834306 | 11 | 1.22E+08 | A | G | 0.014 | 0.003 | 1.96E-08 | 0.42 | *Intron:MIR100HG* | 1166715 |  |
| rs1106363 | 11 | 1.32E+08 | T | C | 0.017 | 0.003 | 9.2E-11 | 0.34 | *Intron:NTM* | 1218288 |  |
| rs2010921 | 11 | 1.32E+08 | A | G | 0.017 | 0.003 | 2.47E-10 | 0.31 | *Intron:NTM* | 1210311 |  |
| rs11057005 | 12 | 16748721 | A | G | 0.016 | 0.003 | 9.12E-10 | 0.56 | *Intron:LMO3* | 1209809 |  |
| rs13906 | 12 | 49952394 | C | T | 0.025 | 0.004 | 1.98E-09 | 0.89 | *Utr3:MCRS1* | 1216874 |  |
| rs4759229 | 12 | 56474480 | G | A | 0.016 | 0.003 | 6.53E-09 | 0.66 | *Intron:ERBB3* | 1225579 |  |
| rs7969559 | 12 | 69655167 | A | G | 0.017 | 0.003 | 1.53E-09 | 0.29 | *Intron:CPSF6* | 1225323 |  |
| rs7134009 | 12 | 75263193 | T | C | 0.016 | 0.003 | 4.3E-08 | 0.71 | *Intergenic* | 1168657 |  |
| rs77215829 | 12 | 1.13E+08 | A | C | 0.024 | 0.004 | 2.02E-10 | 0.87 | *Intron:HECTD4* | 1174145 |  |
| rs1109480 | 12 | 1.21E+08 | G | A | 0.017 | 0.003 | 1.84E-10 | 0.62 | *Intron:CABP1* | 1204679 |  |
| rs11611651 | 12 | 1.33E+08 | A | G | 0.027 | 0.005 | 2.05E-09 | 0.09 | *Intron:GOLGA3* | 1199764 |  |
| rs17197663 | 13 | 38172867 | G | A | 0.022 | 0.004 | 2.06E-08 | 0.88 | *Utr5:POSTN* | 1226280 |  |
| rs4264267 | 13 | 38359676 | T | C | 0.015 | 0.003 | 6.82E-09 | 0.53 | *Intron:TRPC4* | 1207909 |  |
| rs61959481 | 13 | 55834929 | G | A | 0.020 | 0.003 | 7.95E-11 | 0.79 | *Intergenic* | 1194699 |  |
| rs3098272 | 13 | 55931424 | A | C | 0.018 | 0.003 | 2.08E-08 | 0.20 | *Intergenic* | 1227577 |  |
| rs9538162 | 13 | 59265043 | C | T | 0.017 | 0.003 | 1.76E-11 | 0.42 | *Intergenic* | 1228579 |  |
| rs1413119 | 13 | 59339281 | C | T | 0.015 | 0.003 | 4.77E-09 | 0.60 | *Intergenic* | 1224964 |  |
| rs56367474 | 13 | 59454139 | C | T | 0.017 | 0.003 | 4.2E-10 | 0.70 | *Intergenic* | 1222448 |  |
| rs55786907 | 13 | 59871584 | G | A | 0.019 | 0.003 | 1.84E-08 | 0.16 | *Intergenic* | 1224212 |  |
| rs4886207 | 13 | 60705792 | T | C | 0.016 | 0.003 | 8.78E-10 | 0.36 | *Intron:DIAPH3* | 1212549 |  |
| rs9540731 | 13 | 66949370 | C | T | 0.018 | 0.003 | 3.42E-12 | 0.49 | *Intron:PCDH9* | 1226845 |  |
| rs9545155 | 13 | 80191873 | T | C | 0.016 | 0.003 | 3.04E-10 | 0.52 | *Intergenic* | 1227473 |  |
| rs1772572 | 13 | 81191176 | C | A | 0.017 | 0.003 | 5.62E-10 | 0.68 | *Intergenic* | 1226332 |  |
| rs75674569 | 13 | 96823724 | G | A | 0.025 | 0.004 | 2.58E-09 | 0.90 | *Intron:HS6ST3* | 1209629 |  |
| rs7333559 | 13 | 1.01E+08 | G | A | 0.023 | 0.003 | 5.94E-14 | 0.22 | *Intron:CLYBL\|LOC101927437* | 1187406 |  |
| rs1108130 | 13 | 1.01E+08 | A | T | 0.024 | 0.003 | 1.57E-14 | 0.21 | *Exon:LINC00554* | 1198864 |  |
| rs12855717 | 13 | 1.01E+08 | T | C | 0.016 | 0.003 | 1.22E-09 | 0.54 | *Intergenic* | 1215247 |  |
| rs12878369 | 14 | 28346502 | A | C | 0.017 | 0.003 | 1.6E-11 | 0.41 | *Intergenic* | 1222389 |  |
| rs2145451 | 14 | 29316842 | T | C | 0.020 | 0.003 | 5.44E-10 | 0.81 | *Intergenic* | 1162979 | 1 |
| rs9323328 | 14 | 58653514 | A | G | 0.014 | 0.003 | 2.55E-08 | 0.46 | *Intergenic* | 1193275 |  |
| rs1811739 | 14 | 77529375 | A | G | 0.018 | 0.003 | 5.97E-10 | 0.25 | *Intron:LINC02288* | 1201000 |  |
| rs8005334 | 14 | 79563654 | G | T | 0.017 | 0.003 | 3.44E-10 | 0.36 | *Intron:NRXN3* | 1224448 |  |
| rs34940743 | 14 | 80102233 | G | A | 0.016 | 0.003 | 2.8E-09 | 0.35 | *Intron:NRXN3* | 1193949 |  |
| rs2925128 | 14 | 98362355 | T | C | 0.017 | 0.003 | 3.67E-10 | 0.39 | *Intergenic* | 1168620 |  |
| rs1381287 | 14 | 98597552 | T | C | 0.018 | 0.003 | 1.81E-12 | 0.47 | *Intergenic* | 1197898 |  |
| rs55913542 | 14 | 99693843 | T | G | 0.019 | 0.003 | 3.25E-08 | 0.18 | *Intron:BCL11B* | 1217144 |  |
| rs1435672 | 15 | 36399479 | C | T | 0.014 | 0.003 | 3.82E-08 | 0.56 | *Intergenic* | 1226249 |  |
| rs281296 | 15 | 47685010 | A | G | 0.025 | 0.003 | 1.59E-20 | 0.36 | *Intron:SEMA6D* | 1226437 |  |
| rs1435741 | 15 | 47935843 | A | G | 0.018 | 0.003 | 1.09E-12 | 0.43 | *Intron:SEMA6D* | 1227373 | 1 |
| rs56902655 | 15 | 63898709 | T | G | 0.022 | 0.004 | 4.09E-09 | 0.86 | *Intergenic* | 1219705 |  |
| rs2289791 | 15 | 67476952 | G | T | 0.018 | 0.003 | 2.01E-09 | 0.75 | *Intron:SMAD3* | 1192762 |  |
| rs60833441 | 15 | 74048768 | A | G | 0.014 | 0.003 | 2.28E-08 | 0.54 | *Intergenic* | 1222750 |  |
| rs62007780 | 15 | 78025464 | G | T | 0.016 | 0.003 | 7.48E-10 | 0.58 | *Intron:LINGO1* | 1213351 |  |
| rs12442563 | 15 | 83893243 | G | T | 0.023 | 0.003 | 3.13E-14 | 0.78 | *Intergenic* | 1225691 | 1 |
| rs4310804 | 15 | 96858409 | C | G | 0.018 | 0.003 | 7.55E-10 | 0.75 | *Intron:NR2F2-AS1* | 1181540 |  |
| rs8027457 | 15 | 99204101 | C | T | 0.015 | 0.003 | 1.88E-09 | 0.51 | *Intron:IGF1R* | 1222860 |  |
| rs1139897 | 16 | 720986 | G | A | 0.024 | 0.003 | 1.77E-15 | 0.77 | *Nonsynonymous:RHOT2* | 1222828 |  |
| rs11076962 | 16 | 5811367 | C | T | 0.018 | 0.003 | 1.2E-10 | 0.28 | *Intergenic* | 1219040 |  |
| rs7192140 | 16 | 10173748 | T | C | 0.017 | 0.003 | 3.4E-11 | 0.50 | *Intron:GRIN2A* | 1221590 |  |
| rs9922607 | 16 | 17570220 | C | T | 0.022 | 0.003 | 3.42E-12 | 0.80 | *Intergenic* | 1191364 |  |
| rs9941217 | 16 | 18050926 | C | G | 0.019 | 0.003 | 3.5E-12 | 0.65 | *Intergenic* | 1213511 |  |
| rs7188873 | 16 | 24727064 | G | A | 0.020 | 0.003 | 8.46E-15 | 0.61 | *Intron:TNRC6A* | 1222236 |  |
| rs6497840 | 16 | 25351633 | A | G | 0.023 | 0.003 | 2.01E-15 | 0.71 | *Intergenic* | 1164927 |  |
| rs4785187 | 16 | 49766772 | A | G | 0.020 | 0.003 | 6.55E-11 | 0.22 | *Intron:ZNF423* | 1217011 |  |
| rs8050598 | 16 | 49891964 | T | C | 0.019 | 0.003 | 1.76E-10 | 0.25 | *Intergenic* | 1102950 |  |
| rs12918191 | 16 | 50945156 | A | G | 0.020 | 0.003 | 3.14E-11 | 0.76 | *Intergenic* | 1216229 |  |
| rs9302604 | 16 | 69576894 | G | A | 0.019 | 0.003 | 3.29E-13 | 0.44 | *Intergenic* | 1224382 |  |
| rs9936784 | 16 | 72230694 | G | T | 0.014 | 0.003 | 4.33E-08 | 0.53 | *Intergenic* | 1221890 |  |
| rs62052916 | 16 | 72574550 | A | T | 0.032 | 0.005 | 1.62E-10 | 0.93 | *Intron:LINC01572* | 1221109 |  |
| rs4788676 | 16 | 72950468 | T | C | 0.018 | 0.003 | 4.92E-09 | 0.77 | *Intron:ZFHX3* | 1222092 |  |
| rs61537885 | 16 | 75620118 | T | C | 0.040 | 0.007 | 8.06E-09 | 0.96 | *Intergenic* | 935451 | 1 |
| rs117657830 | 16 | 75766873 | A | G | 0.038 | 0.006 | 3.18E-09 | 0.96 | *Intergenic* | 1178553 |  |
| rs1050847 | 16 | 87443734 | C | T | 0.015 | 0.003 | 7.37E-09 | 0.44 | *Utr3:ZCCHC14* | 1210408 |  |
| rs11642231 | 16 | 89608702 | G | A | 0.016 | 0.003 | 3.44E-09 | 0.63 | *Intron:SPG7* | 1213205 |  |
| rs4790874 | 17 | 1995177 | T | C | 0.017 | 0.003 | 8.43E-12 | 0.53 | *Intron:SMG6* | 1199145 |  |
| rs11078713 | 17 | 7795972 | A | G | 0.015 | 0.003 | 1.59E-08 | 0.58 | *Intron:CHD3* | 1211948 |  |
| rs28441558 | 17 | 7803118 | T | C | 0.036 | 0.006 | 1.24E-10 | 0.94 | *Intron:CHD3* | 1199036 |  |
| rs11651955 | 17 | 16235462 | G | A | 0.014 | 0.003 | 3.74E-08 | 0.50 | *Intergenic* | 1221896 |  |
| rs67777803 | 17 | 27323322 | G | T | 0.025 | 0.003 | 3.18E-13 | 0.83 | *Intron:SEZ6* | 1224242 |  |
| rs2344976 | 17 | 30685935 | T | C | 0.015 | 0.003 | 7.98E-09 | 0.39 | *Intron:ZNF207* | 1226520 |  |
| rs3764351 | 17 | 37824339 | G | A | 0.015 | 0.003 | 3.89E-08 | 0.34 | *Intron:PNMT* | 1216758 |  |
| rs72836318 | 17 | 44121579 | T | C | 0.017 | 0.003 | 7E-09 | 0.75 | *Intron:KANSL1* | 1140175 | 1 |
| rs17692129 | 17 | 44793283 | T | C | 0.020 | 0.003 | 4.57E-13 | 0.33 | *Intron:NSF* | 1194121 |  |
| rs75919030 | 17 | 50193197 | T | C | 0.021 | 0.003 | 3.35E-13 | 0.73 | *Intron:CA10* | 1216083 |  |
| rs2938134 | 17 | 50243397 | C | A | 0.018 | 0.003 | 3.14E-10 | 0.33 | *Intergenic* | 1161698 |  |
| rs2587507 | 17 | 77790135 | T | C | 0.015 | 0.003 | 8.69E-09 | 0.50 | *Intergenic* | 1226474 |  |
| rs34342129 | 18 | 5872472 | T | C | 0.014 | 0.003 | 2.13E-08 | 0.49 | *Intergenic* | 1226679 |  |
| rs4476253 | 18 | 25253297 | G | A | 0.018 | 0.003 | 5.78E-10 | 0.76 | *Intergenic* | 1212112 |  |
| rs7505855 | 18 | 31696075 | C | T | 0.017 | 0.003 | 5.31E-11 | 0.41 | *Intron:NOL4* | 1219699 |  |
| rs8096225 | 18 | 36921851 | C | A | 0.016 | 0.003 | 2.63E-08 | 0.70 | *Intron:MIR924HG* | 1224058 |  |
| rs67050670 | 18 | 39297254 | A | G | 0.020 | 0.003 | 2.34E-11 | 0.77 | *Intergenic* | 1218972 |  |
| rs2359180 | 18 | 41314171 | A | G | 0.014 | 0.003 | 4.98E-08 | 0.63 | *Intergenic* | 1056112 |  |
| rs72898831 | 18 | 42658643 | A | G | 0.024 | 0.004 | 4.14E-12 | 0.85 | *Intergenic* | 1217375 |  |
| rs8083764 | 18 | 49874515 | G | T | 0.016 | 0.003 | 7.97E-09 | 0.69 | *Intron:DCC* | 1229740 |  |
| rs1373178 | 18 | 49967811 | T | G | 0.020 | 0.003 | 4.16E-15 | 0.41 | *Intron:DCC* | 1203036 |  |
| rs62098013 | 18 | 50863861 | A | G | 0.018 | 0.003 | 2.24E-11 | 0.37 | *Intron:DCC* | 1165372 |  |
| rs72938304 | 18 | 53661743 | G | A | 0.027 | 0.004 | 1.36E-11 | 0.89 | *Intergenic* | 1180798 |  |
| rs11872397 | 18 | 72535282 | G | A | 0.017 | 0.003 | 5.2E-09 | 0.75 | *Intron:ZNF407* | 1200662 |  |
| rs71367544 | 18 | 77574374 | T | C | 0.021 | 0.003 | 8.54E-11 | 0.20 | *Intergenic* | 1219600 |  |
| rs76608582 | 19 | 4474725 | C | A | 0.035 | 0.006 | 4.88E-09 | 0.95 | *Intron:HDGFL2* | 1044654 |  |
| rs10853981 | 19 | 4965064 | A | G | 0.015 | 0.003 | 4.88E-08 | 0.33 | *Intergenic* | 1204615 |  |
| rs113230003 | 19 | 18460956 | G | A | 0.019 | 0.003 | 1.05E-10 | 0.75 | *Intron:PGPEP1* | 1165454 |  |
| rs8103660 | 19 | 18566395 | C | T | 0.016 | 0.003 | 3.03E-09 | 0.35 | *Intron:ELL* | 1199098 |  |
| rs117734003 | 19 | 51129745 | C | G | 0.030 | 0.005 | 2.57E-09 | 0.07 | *Intron:SYT3* | 1095038 |  |
| rs1126757 | 19 | 55879872 | T | C | 0.014 | 0.003 | 2.92E-08 | 0.47 | *Synonymous:IL11* | 1211443 |  |
| rs6050446 | 20 | 25195509 | G | A | 0.054 | 0.008 | 8.8E-13 | 0.97 | *Nonsynonymous:ENTPD6* | 1144803 |  |
| rs6058782 | 20 | 29946968 | T | C | 0.030 | 0.004 | 1.78E-11 | 0.91 | *Intergenic* | 1198733 | 1 |
| rs1555445 | 20 | 31175258 | T | A | 0.019 | 0.003 | 7.75E-12 | 0.32 | *Upstream:NOL4L-DT* | 1190155 |  |
| rs6073075 | 20 | 42015801 | T | A | 0.019 | 0.003 | 2.44E-08 | 0.18 | *Intergenic* | 1192887 |  |
| rs910912 | 20 | 54462393 | T | C | 0.017 | 0.003 | 7.82E-09 | 0.26 | *Intergenic* | 1223801 |  |
| rs6011779 | 20 | 61984317 | C | T | 0.019 | 0.003 | 2.83E-09 | 0.19 | *Intron:CHRNA4* | 1186828 |  |
| rs3810496 | 20 | 62406886 | C | T | 0.016 | 0.003 | 1.54E-09 | 0.62 | *Intron:ZBTB46* | 1142447 |  |
| rs4818005 | 21 | 40588819 | G | A | 0.020 | 0.003 | 1.09E-14 | 0.42 | *Intron:BRWD1* | 1169026 |  |
| rs139896 | 22 | 38397797 | C | T | 0.015 | 0.003 | 7.14E-09 | 0.65 | *Intron:POLR2F* | 1228133 |  |
| rs4822102 | 22 | 42698430 | C | T | 0.017 | 0.003 | 2.78E-10 | 0.38 | *Intergenic* | 1227249 |  |
| rs9627272 | 22 | 46442288 | G | C | 0.015 | 0.003 | 2.42E-09 | 0.59 | *Intergenic* | 1194396 |  |

Chr indicates chromosome; EA, effect allele; EAF, effect allele frequency; NEA, non-effect allele; SNP, single-nucleotide polymorphisms. N presents the effective sample size for each SNP in the original GWAS.

^a^ These SNPs were excluded due to linkage disequilibrium at R^2^>0.1 based on 1000 genomes data confined to European ancestry.

**Supplementary Table 2.** Definition of psychiatric disorders included in the present MR study

| **Psychiatric disorder** | **Definition** |
| --- | --- |
| Suicide attempts | Suicide attempts were identified by screening the Danish Psychiatric Central Research Register and the National Registry of Patients for diagnoses of suicide attempts (ICD-10: X60–X84). In addition, contacts where the “reasons for contact”-variable indicated suicide attempt were included, as well as combinations of diagnoses where the main diagnosis had been recorded as a mental disorder (ICD-10: F chapter) together with a diagnosis of poisoning by drugs or other substances (ICD-10: T36–T50, T52–T60) or injuries to hand, wrist, and forearm (ICD-10: S51, S55, S59, S61, S65, S69). |
| Post-traumatic stress disorder | All patients came from clinically deeply characterized and small patient groups to large cohorts with self-reported PTSD symptoms. |
| Schizophrenia | The cases were chosen by using fundamentally different ascertainment methods (e.g. hospital discharge register records or patients defined as having treatment-resistant schizophrenia and registered to use the medication Clozapine). |
| Bipolar disorder | Cases were required to meet international consensus criteria (DSM-IV, ICD-9, or ICD-10) for a lifetime diagnosis of BD established using structured diagnostic instruments from assessments by trained interviewers, clinician-administered checklists, or medical record review. |
| Major depressive disorder | Depressive symptoms were measured with the CES-D scale (10-item version [CHS, NHS, Rush MAP, Rush ROS], 11- item version [ARIC1], or 20-item version [ARIC2, BLSA, ERF, FHS, HBCS, Health ABC, InCHIANTI, MESA, RS-I, RS-II, RS-III, SardiNIA]). |
| Insomnia | Participants completed one or more questions related to seven phenotypic concepts concerning sleep. Insomnia cases affirmed at least one of the following questions:  "Have you ever been diagnosed with, or treated for: Insomnia?"; “Have you ever been diagnosed with, or treated for, any of the following conditions: Insomnia but not Narcolepsy, Sleep apnea or Restless leg syndrome”, etc. |
| Anxiety | Standardized assessment instruments were used to generate DSM-based AD diagnoses, with some exceptions. Five core anxiety included in studies: GAD, PD, social phobia, agoraphobia, and specific phobias. |

**Supplemental Table 3.** Heterogeneity in the association between smoking initiation and psychiatric disorders

| **Psychiatric disorder** | **I^2^** | **Cochrane Q** | ***p* ^a^** | **Rucker’s Q** | ***p* ^b^** |
| --- | --- | --- | --- | --- | --- |
| Suicide attempts | 19 | 376 | 0.003 | 374 | 0.003 |
| Post-traumatic stress disorder | 4 | 364 | 0.296 | 364 | 0.283 |
| Schizophrenia | 68 | 1099 | ＜0.001 | 1093 | ＜0.001 |
| Bipolar disorder | 52 | 736 | ＜0.001 | 735 | ＜0.001 |
| Major depressive disorder | 70 | 1171 | ＜0.001 | 1164 | ＜0.001 |
| Insomnia | 54 | 744 | ＜0.001 | 742 | ＜0.001 |
| Anxiety | 7 | 371 | 0.178 | 371 | 0.169 |

Cochrane Q, Cochrane Q statistic; Rucker’s Q, Rucker’s Q statistic.

^a^ *P* for I^2^ and Cochrane Q values.

^b^ *P* for Rucker’s Q

**Supplementary Table 4**. The causal effect of depression on smoking initiation

| **Method** | **OR** | **95% CI** | **P** |
| --- | --- | --- | --- |
| IVW-random effects | 1.11 | 1.02-1.22 | 0.016 |
| Weighted median | 1.19 | 1.09-1.30 | <0.001 |
| MR-Egger | 0.83 | 0.56-1.24 | 0.360 |

There was a moderate heterogeneity among used SNPs (I^2^=62%) and no pleiotropy was detected by MR-Egger (the intercept=0.007; *p*=0.138).
